# Shaping death: how the microbiome regulates tumour cell demise and therapy response

**DOI:** 10.1007/s10555-026-10318-1

**Published:** 2026-02-20

**Authors:** Martina Raudenska, Jan Balvan, Eliska Zgarbova, David Kalfert, Jan Plzak, Michal Masarik

**Affiliations:** 1 Department of Pathological Physiology, Faculty of Medicine, Masaryk University, Kamenice 5, 625 00 Brno, Czech Republic; 2 Department of Physiology, Faculty of Medicine, Masaryk University, Kamenice 5, 625 00 Brno, Czech Republic; 3 International Clinical Research Center, St. Anne’s University Hospital, Brno, 60200 Czech Republic; 4 Department of Otorhinolaryngology, Head and Neck Surgery, First Faculty of Medicine, Charles University and University Hospital Motol and Homolka, V Uvalu 84, 15006 Prague, Czech Republic; 5 First Faculty of Medicine, Institute of Pathophysiology, Charles University, U Nemocnice 5, 128, 53 Prague, Czech Republic; 6 First Faculty of Medicine, BIOCEV, Charles University, Prumyslova 595, 25250 Vestec, Czech Republic

**Keywords:** Cell death, Apoptosis, Ferroptosis, Microbial metabolites, Cancer metabolism, Cancer therapy, Microbiome

## Abstract

Cell death is a fundamental process that maintains tissue homeostasis and shapes the tumour microenvironment. Cancer cells often evade or reprogram cell death pathways, which leads to malignancy and therapy resistance. On the other hand, the cell death of non-malignant cells significantly influences the adverse effects of anticancer therapy, such as the onset of mucositis or enteritis. Emerging evidence identifies both the tumour and gut microbiomes as critical modulators of these processes. In turn, cell death reshapes the microbial ecosystem by altering nutrient landscapes and immune signalling. Although both cell death and the microbiome are well-studied in cancer, their intersection remains underexplored. This review article addresses this gap by summarising how microbes and microbial metabolites modulate cancer cell death pathways and shape responses to anticancer therapy. We integrated current knowledge on this complex interplay, focusing on key metabolic mediators that can influence cell fate decisions. Understanding this bidirectional crosstalk offers new opportunities to “preserve the good and eliminate the bad” within the tumour–microbiome axis, paving the way for precision microbiome-informed cancer therapies.

## Introduction

Cell death serves as a crucial mechanism for maintaining physiological homeostasis by eliminating damaged or unnecessary cells, but it can also arise as a response to harmful stimuli. In cancer, the ability of cancer cells to evade or reprogram cell death pathways is central to malignancy [1]. Cell death is a fundamental process that further shapes the tumour microenvironment (TME), influencing tumour growth, immune activation, and therapeutic response [2, 3]. Recent studies have revealed that, beyond genetic and stromal determinants, the microbiome also plays a role in modulating cell death pathways. Microbial metabolites can directly or indirectly regulate cell death pathways in both cancer and TME cells. Anticancer therapies add further complexity as they trigger cell death while also altering the composition of the microbiome, microbial metabolism, and the favourable or unfavourable conditions for the growth of different bacterial species. For example, radiation therapy can alter short-chain fatty acid (SCFA)-producing bacteria, leading to changes in SCFA levels that may negatively impact cancer treatment outcomes [4]. Specifically, a reduced ratio of *Bacteroidetes* to *Firmicutes* after radiotherapy significantly affects the lipid profile of the intestinal epithelial barrier, leading to death of intestinal cells and progression of enteritis [5]. Chemoradiotherapy induces death of intestinal epithelial cells through various pathways, including apoptosis, necroptosis, and pyroptosis, leading to compromised gut barrier integrity and inflammation [6, 7]. Surprisingly, a recent study has shown that intestinal low-dose irradiation (ILDR, 1–3 Gy) induces an abscopal effect via distal immunostimulation, primarily through microbiota-driven alterations in systemic metabolism. ILDR modulates cholesterol and bile acid pathways, thereby enhancing the antigen-presenting capacity of dendritic cells (DCs). Elevated cholesterol accumulation in DCs further promotes antigen presentation and CD8⁺ T-cell activation without exhaustion. Certain *Christensenella minuta* strains further boost ILDR and PD-L1 blockade by promoting migration of intestinal dendritic cells to tumour-draining lymph nodes. A phase II trial confirms that ILDR can overcome immunotherapy resistance [8].

Microbiome-derived signals can modulate tumour cell death pathways, changing cancer cells' sensitivity to therapy, while tumour cells and their death reshape the local microbial community by changing nutrient and immune landscapes. Dying mammalian cells produce and shed metabolites that bacteria, such as *Salmonella* and *E. coli*, use as fuel to assist them in colonisation during inflammatory conditions or chemotherapy-induced mucositis [9]. Lung adenocarcinoma cells also create selective pressure favouring bacteria capable of synthesising L-methionine, an essential amino acid they cannot produce themselves. This mutualistic interaction enables bacteria to supply methionine to nutrient-deprived tumours and stop them from dying by ferroptosis [10], while cancer cells, in turn, promote the growth of methionine-producing bacteria [11]. Deciphering the bidirectional interplay between the microbiome and cell death regulation may provide a new therapeutic angle: leveraging microbiome modulation to promote immunogenic cell death pathways in tumours, while limiting resistance mechanisms. Such insights could guide the development of microbiome-based adjuvants to enhance standard therapies. Although cell death and microbiome are extensively studied in the context of cancer, the intersection between the two remains insufficiently explored. This review article aims to bridge the critical knowledge gap by providing a comprehensive overview of the current understanding of how microbial metabolites interact with cancer cell death pathways and influence responses to anticancer therapies.

### Basic overview of regulated cell death pathways

The various forms of regulated cell death can be categorised into three distinct groups: suicide, sabotage, and murder. Suicide refers to forms of cell death that occur via molecular pathways evolved in response to signals. While these signals are not necessarily destructive in themselves, under certain circumstances, they can lead to cell death. Sabotage involves cell death that occurs when an active molecular process necessary for normal cell function becomes nonfunctional. Such forms of sabotage are autophagy-related cell death, cuproptosis, parthanatos, or ferroptosis. Murder involves cell death caused by another cell whose task is to kill the target cell. Within this broad framework, cells that die by apoptosis, pyroptosis, or necroptosis are said to commit suicide. Those that die by cytotoxic lymphocytes, antibody-dependent cytotoxicity, or complement-mediated lysis are murdered [12] (see Table 1).

**Table 1:** Regulated Cell Death Categories (Based on Green [12])

Group	Definition	Cell Death Type	Short Description
Suicide	Cells activate internally programmed death in response to internal or external signals that are not directly destructive but can trigger cell death pathways	Apoptosis	Apoptosis occurs via the intrinsic pathway (triggered by internal stress through mitochondria) or the extrinsic pathway (triggered by external death‑receptor signals); usually involves caspases, DNA fragmentation, and cell shrinkage
		Pyroptosis	Inflammatory cell death triggered by inflammasomes; involves gasdermin pores and cell swelling/lysis
		Necroptosis	Regulated necrotic death mediated by RIPK1–RIPK3–MLKL; results in membrane rupture and inflammation
		NETosis	Regulated form of cell death in neutrophils. Neutrophils expel chromatin and granular proteins to form neutrophil extracellular traps (NETs) that immobilise and kill microbes; involves unique chromatin decondensation and lytic rupture
Sabotage	Cell death occurs because a normally essential process becomes dysfunctional, leading to the collapse of homeostasis	Autophagy-related cell death	Excessive autophagy leads to self-digestion and loss of essential cellular components
		Cuproptosis	Copper-induced cell death driven by disruption of mitochondrial metabolism and protein aggregation
		Parthanatos	PARP1-dependent death caused by massive DNA damage and NAD⁺/ATP depletion; AIF translocation to the nucleus
		Ferroptosis	Iron-dependent lipid peroxidation overwhelms antioxidant defence, causing membrane damage
Murder	An external cell or system kills the target cell	Cytotoxic lymphocyte–mediated killing	Killer T-cells or NK-cells induce death via perforin–granzymes or death receptors
		Antibody-dependent cellular cytotoxicity	Antibodies bind the target; immune effector cells recognise Fc-fragments and induce cell death
		Complement-mediated lysis	Complement activation forms a membrane attack complex that punches holes in the target cell membrane

### Cell suicide

The best-known form of cell suicide is apoptosis. Apoptosis is usually driven by caspase activation (however, caspase-independent apoptosis was also observed). Apoptosis occurs via two main routes: the intrinsic pathway, controlled by mitochondrial outer membrane permeabilization (MOMP), regulated by Bcl-2 family proteins, and the extrinsic pathway, triggered by death receptors [13]. Apoptotic cells undergo nuclear condensation, DNA fragmentation, and membrane blebbing, culminating in the formation of apoptotic bodies that are usually cleared by efferocytosis. The intrinsic apoptotic pathway is triggered by stress signals within the cell, such as DNA damage, oxidative stress, or growth factor deprivation. These signals activate pro-apoptotic proteins (e.g., BAX and BAK) from the Bcl-2 family, causing MOMP. This releases cytochrome c into the cytosol, where it forms an apoptosome complex with APAF-1 and pro-caspase-9. This leads to the activation of caspase-9 and subsequent executioner caspases, such as caspase-3; consequently, the cleaved Poly ADP-ribose polymerase (PARP) is formed. In the absence of anti-apoptotic proteins, activators such as BID, BIM, and PUMA can directly bind to and activate BAX and BAK. Additionally, sensitizers such as BAD, NOXA, BIK, and PUMA can suppress the inhibitory effect of BCL-2, BCL-XL, and MCL-1 on BAX and BAK, thereby indirectly activating them [14].

The extrinsic pathway of apoptosis is initiated by extrinsic ligands (e.g., FasL, TNF-α, and TRAIL) binding to death receptors (e.g., Fas/CD95 and TNFR1) on the cell surface. This leads to the formation of a death-inducing signalling complex (DISC), which activates the initiator caspase-8. Caspase-8 then activates the executor caspases, and the cleaved PARP is formed [14]. The extrinsic apoptotic pathway, which is controlled by death receptors, may proceed differently in different cell types. In adult lymphocytes or thymocytes (lymphocytes that originate in the thymus), i.e., cells that we refer to as cell type I, the proteolytic maturation of executor caspases, which is mediated by high levels of activated caspase-8, is usually sufficient to activate cell death. Therefore, apoptosis occurs without the involvement of mitochondria. In these cells, apoptosis cannot be inhibited by increased expression of anti-apoptotic Bcl-2 family proteins, or by the simultaneous deletion of BAX and BAK1, or the loss of BID [15, 16]. In type II cells (e.g., hepatocytes, pancreatic β-cells, and most tumour cells), the external signal alone is insufficient to activate enough caspase-8 to trigger apoptosis. The activation of caspases-3 and −7 is prevented in type II cells by the XIAP protein [17], and thus the extrinsic apoptotic pathway also requires the proteolytic cleavage of BID for successful activation [18]. The cleaved form of BID (tBID), which is produced in this process, is transported to the outer mitochondrial membrane. There, it acts on BAX/BAK-dependent mitochondrial outer membrane permeabilisation (MOMP) and subsequent apoptosome assembly, which is accompanied by caspase-9 activation. While basal XIAP levels are similar in type I (thymocyte) and type II (hepatocyte) cells, stimulation of the FAS receptor leads to a rapid decrease in XIAP levels in thymocytes, but an increase in hepatocytes [17, 19]. Therefore, it can be concluded that type I cells undergo apoptosis independently of mitochondria, whereas type II cells undergo MOMP.

Necroptosis is primarily initiated via death receptors, such as TNFR1. The most widely studied form to date is TNF-α-induced necroptosis, but many other stimuli capable of activating necroptosis have been identified. These include CD95L (also known as FASL), TRAIL, TWEAK, genotoxic stress, polyclonal T-cell receptor stimulation, viral activation of Z-DNA binding protein 1 (ZBP1, also known as DAI), and certain anticancer drugs, such as shikonin and GX15-070 [20]. During necroptosis, RIPK1 becomes activated and interacts with RIPK3 to form a multiprotein complex known as the necrosome. Within the necrosome, RIPK3 phosphorylates the pseudokinase MLKL, which then forms oligomers and moves to the plasma membrane. There, phosphorylated MLKL disrupts the membrane's integrity, leading to cell swelling and ultimately lysis. Necroptotic cells release damage-associated molecular patterns (DAMPs), stimulating immune responses. The interaction of active MLKL with the plasma membrane requires certain inositol phosphate forms generated by lipid kinases (e.g., IP5 and IP6); without these, necroptosis either does not occur or is reduced [12].

Pyroptosis is mostly triggered by intracellular pattern recognition receptors (PRRs) detecting pathogenic microorganisms or dangerous signals. This leads to the formation of a multiprotein complex called an inflammasome, which activates caspase-1. The best-characterised proteins in inflammasomes are NLRP1, NLRP3, NLRC4, and AIM2 [21]. Activated caspase-1 then cleaves gasdermin D (GSDMD). The N-terminal fragment of GSDMD then inserts itself into the plasma membrane, forming pores. GSDMD pores favour ninjurin 1 (NINJ1)-induced plasma membrane rupture and cell death [22]. This leads to cell swelling, lysis, and the release of inflammatory interleukins (IL-1β and IL-18), amplifying immune signalling. In some contexts, pyroptosis can also be initiated by caspase-4 or −5 (in humans) or caspase-11 (in mice) in response to intracellular lipopolysaccharide (LPS) during infection with gram-negative bacteria, leading to non-canonical inflammasome activation [23]. Under certain circumstances, the N-terminal domain of GSDMD can be translocated to the outer mitochondrial membrane, causing permeabilisation independently of BAX and BAK [24]. Damaged mitochondria may further facilitate the formation of NLRP3 inflammasomes, as destabilised mitochondria induce cardiolipin externalisation, which promotes NLRP3 inflammasome assembly and pyroptosis initiation [25].

NETosis is a regulated form of cell death in neutrophils that helps defend the host against pathogens. It is initiated when neutrophils are activated by microbes, proinflammatory cytokines, or chemical stimuli. This triggers a cascade involving reactive oxygen species (ROS), which drive chromatin decondensation. Neutrophil elastase (NE) translocates to the nucleus and cleaves histones, while myeloperoxidase (MPO) aids chromatin relaxation and nuclear envelope breakdown. The nuclear and granular contents then mix, and the plasma membrane ruptures, releasing neutrophil extracellular traps (NETs) composed of chromatin and histones. These NETs capture and kill pathogens, limiting their spread. Many bacteria trigger NET formation, or NETosis, including species like *Staphylococcus aureus*, *Klebsiella pneumoniae*, *Fusobacterium nucleatum*, and *Mycobacterium bovis* [26]. The mechanism can involve direct detection of bacteria or recognition of bacterial byproducts, including lactate, which signals the release of NETs [27]. NETs protect cancer cells by forming a physical barrier around them, shielding tumour cells from cytotoxic T-lymphocytes and NK-cells by masking their interaction sites. Additionally, NETs promote metastasis by enhancing tumour cell migration and invasion [28]. Nuclease expression by *Staphylococcus aureus* facilitates degradation of NETs [29].

### Cell sabotage

Many of the newly discovered forms of cell death can be categorised as sabotage. While these are active forms of cell death, they did not evolve primarily for the purpose of killing cells. One example of a "sabotage" type of cell death is ferroptosis, which occurs when the mechanisms necessary to prevent or detoxify lipid peroxides in membranes fail. Unlike apoptosis or necroptosis, ferroptosis does not involve the activation of caspases. Instead, it is driven by the accumulation of ROS, which specifically oxidise polyunsaturated fatty acids (PUFAs) in cell membranes. Lipid peroxidation of the PUFAs occurs via iron-dependent Fenton reactions or enzymatic pathways involving lipoxygenases, cyclooxygenases, and cytochrome P450, producing hydroperoxides that amplify ferroptotic signalling. Enzymes such as acyl-CoA synthetase long chain family member 4 (ACSL4) and lysophosphatidylcholine acyltransferase 3 (LPCAT3) incorporate PUFAs into membrane phospholipids, thereby increasing cellular susceptibility to ferroptosis. Several key mechanisms, including iron metabolism, the mode of lipid peroxidation, and antioxidant defence, tightly regulate this process. The enzyme glutathione peroxidase 4 (GPX4) plays a central protective role, reducing lipid hydroperoxides to non-toxic lipid alcohols with the help of glutathione (GSH) [30]. Numerous studies have suggested that inducing ferroptosis could affect the effectiveness of tumour treatment and even reverse treatment resistance [31]. The levels of intracellular iron and iron uptake are significantly higher in rapidly proliferating cells, such as tumour cells. Furthermore, tumour cells often exhibit abnormal lipid metabolism and increased ROS production. These factors sensitise malignant cells to ferroptosis. However, cancer cells counter this problem by increasing the activity of ferroptosis-inhibiting mechanisms, such as GPX4. Therefore, ferroptosis inducers, including GPX4 inhibitors, are somewhat specific in their targeting of cancer cells [32]. Some chemotherapeutic agents used to treat solid tumours are likely to trigger ferroptosis. For instance, altretamine, a DNA-alkylating agent commonly employed in the treatment of late-stage ovarian cancer, induces ferroptosis by inhibiting GPX4. Recently, sulfasalazine, a non-steroidal anti-inflammatory drug traditionally used to treat rheumatoid arthritis, has been shown to induce ferroptosis in head and neck tumours. Sorafenib, a multikinase inhibitor (VEGFR, PDGFR, c-Kit and RET) used to treat hepatocellular carcinoma, can also induce ferroptosis [33].

Microbes can influence ferroptosis by altering host iron metabolism. Many bacteria found in the TME secrete siderophores that scavenge extracellular iron, depriving host cells of iron needed for lipid peroxidation and thereby limiting ferroptosis. These bacteria include *Pseudomonas aeruginosa*, *Escherichia coli*, and *Staphylococcus spp*., which produce siderophores like pyoverdine (from *P. aeruginosa*), enterobactin (from *E. coli*), and staphyloferrin A (from *Staphylococcus* spp.) to acquire essential iron for their survival and growth within the tumour [34, 35]. Probiotics, such as *Lactobacillus plantarum,* can inhibit ferroptosis by releasing specific extracellular vesicles [36] and reducing oxidative stress [37]. Some studies show that gut microbiota–derived metabolites can regulate ferroptosis via the GPX4/Nrf2 pathway. For example, 3-hydroxyphenylacetic acid and capsiate upregulate GPX4 to inhibit ferroptosis and protect against aging-related spermatogenic dysfunction or intestinal ischemia reperfusion injury [38, 39]. Conversely, dysbiosis with harmful bacteria like adherent-invasive *E. coli* reduces GPX4 and ferritin, promoting lipid peroxidation and ferroptosis in intestinal epithelial cells [40]. *P. aeruginosa* promotes ferroptosis by degrading the key anti-ferroptotic enzyme GPX4 via chaperone-mediated autophagy, while simultaneously increasing the labile iron pool and driving ROS-dependent lipid peroxidation [41]. Thus, microbial control of iron availability can either suppress or enhance ferroptotic cell death, shaping infection outcomes and potentially influencing cancer progression.

Another type of 'sabotage' cell death is parthanatos. This type of cell death is primarily caused by the excessive activation of an enzyme called poly(ADP-ribose) polymerase-1 (PARP-1). PARP-1 is normally activated in response to DNA damage to facilitate its repair, but excessive damage can lead to hyperactivation of the enzyme. This results in the depletion of cellular NAD⁺ and ATP, as well as the disruption of metabolic homeostasis. A distinctive feature of parthanatos is the accumulation of poly(ADP-ribose) (PAR) polymers, which are transported from the nucleus to the mitochondria, where they promote the release of apoptosis-inducing factor (AIF). Once released, AIF moves to the nucleus, where it induces extensive DNA fragmentation and chromatin condensation independently of caspases [42]. Bacteria can promote parthanatos through several interconnected mechanisms. Pathogens such as *Helicobacter pylori* and *Escherichia coli* produce genotoxins (e.g., CagA, VacA, colibactin) that induce DNA damage and drive PARP1 hyperactivation [43, 44]. In parallel, NADase (an enzyme that consumes NAD⁺) from *Streptococcus pyogenes*, directly depletes cellular NAD⁺ pools, sensitising host cells to PARP1-dependent energy collapse [45]. This mechanism can contribute to significant oncolytic (cancer-killing) activity of *Streptococcus pyogenes*, which was observed in various cancers, including glioma, hepatoma, and pancreatic cancer [46].

Cell death types classified as "sabotage" also include cuproptosis and necrosis controlled by mitochondrial permeability transition (MPT-controlled necrosis). Cuproptosis is a form of RCD caused by intracellular accumulation of copper ions and is closely linked to mitochondrial metabolism. It occurs when excess copper binds directly to lipoylated components of the Krebs cycle in mitochondria, leading to protein aggregation and proteotoxic stress. Cells with high mitochondrial activity and dependence on oxidative phosphorylation are particularly susceptible to cuproptosis. The discovery of cuproptosis has opened up new possibilities for potential therapy, particularly in tumours with high mitochondrial respiration, by exploiting their sensitivity to copper-induced toxicity [47]. MPT-mediated necrosis is controlled by a change in mitochondrial permeability and is initiated by severe oxidative stress and increased cytosolic Ca^2+^ concentration. A sudden loss of inner mitochondrial membrane permeability causes a collapse in mitochondrial membrane potential and inhibits ATP synthesis. It also triggers a profound ionic imbalance, which leads to the osmotic breakdown of organelles and cell death [48].

### Autophagy between cytoprotection and cell demise

Autophagy is a highly conserved catabolic process that maintains cellular homeostasis by degrading and recycling cytoplasmic components via the lysosomal pathway. The ULK1 complex (comprising ULK1/2, ATG13, FIP200, and ATG101) is responsible for initiating its molecular mechanism, which is typically activated under conditions of nutrient deprivation or stress, when the energy sensor AMPK (AMP-activated protein kinase) is activated, and the growth regulator mTORC1 is inhibited. This triggers the recruitment of the class III PI3K complex I (Beclin 1, VPS34, VPS15, ATG14L), which generates phosphatidylinositol 3-phosphate (PI3P) at the nascent phagophore, allowing the recruitment of downstream ATG proteins. Expansion of the phagophore is driven by ubiquitin-like conjugation systems that coordinate the activity of ATG proteins together with LC3 (microtubule-associated protein 1 light chain 3) and GABARAP family members. The ATG12–ATG5–ATG16L1 complex enhances the final connection of phosphatidylethanolamine (PE) molecules, resulting in the formation of membrane-bound LC3-II and/or GABARAP-PE. This process drives membrane elongation and closure, producing the autophagosome. This double-membrane vesicle engulfs damaged organelles, protein aggregates, or surplus cytoplasm, and subsequently fuses with lysosomes, where lysosomal hydrolases degrade the contents. In addition to managing autophagy induction in complex I, VPS34-Beclin1 also has a role in the fusion of autophagosomes with lysosomes as complex II. Metabolites (amino acids, fatty acids, nucleotides) obtained by autophagy are recycled to sustain ATP production and biosynthesis, especially under stress conditions such as nutrient deprivation, hypoxia, infection, or therapeutic insult [49].

Microbes can modulate autophagy in cancer cells, influencing tumour progression and therapy resistance. For example, *Porphyromonas gingivalis* induces autophagy in oral cancer cells [50]. *Fusobacterium nucleatum* promotes chemoresistance in colorectal and oesophageal cancers by upregulating autophagy-related proteins (ULK1, ATG7, LC3, Beclin1) and enhancing epithelial–mesenchymal transition (EMT) [51–53]. Knockdown of ATG7 reverses this resistance, highlighting its key role [52]. *Salmonella typhimurium* strains (A1-R, VNP20009) trigger autophagy as a host defence mechanism. Blocking autophagy (via ATG5/Beclin1 knockdown or autophagy inhibitors, such as chloroquine and bafilomycin A1) enhances bacterial-mediated cancer cell killing [54]. Meanwhile, cytolethal distending toxin (CDT) produced by *Campylobacter jejuni* suppresses irradiation-induced autophagy by reducing acidic vesicular organelle formation, increasing radiosensitivity in prostate cancer [55].

Mitophagy is a selective form of autophagy that ensures mitochondrial quality control by removing damaged or superfluous mitochondria, thereby maintaining cellular homeostasis and metabolic balance. The process is initiated when dysfunctional mitochondria lose their membrane potential, a signal that recruits key regulatory proteins to the outer mitochondrial membrane. The best-characterised pathway involves the serine/threonine kinase PINK1 (PTEN-induced kinase 1) and the E3 ubiquitin ligase Parkin. Under normal conditions, PINK1 is imported into healthy mitochondria and rapidly degraded. However, upon mitochondrial depolarisation, PINK1 accumulates on the outer mitochondrial membrane, where it phosphorylates ubiquitin and Parkin, leading to Parkin activation. Activated Parkin ubiquitinates multiple outer mitochondrial membrane proteins, creating a signal for the recruitment of autophagy adaptors such as p62/SQSTM1, NBR1, OPTN, and NDP52, which bridge ubiquitinated mitochondria with the autophagy machinery through their LC3-interacting regions. In parallel, Parkin-independent pathways also exist, involving receptors such as BNIP3, NIX, and FUNDC1, which reside on the mitochondrial membrane and directly interact with LC3 via LC3-interacting regions (LIR motifs), bypassing ubiquitination. Ultimately, the isolation membrane, or phagophore, engulfs the damaged mitochondrion, forming a mitophagosome that fuses with lysosomes for degradation. Through these mechanisms, mitophagy plays a dual role: under physiological conditions, it preserves mitochondrial function and prevents oxidative stress, while under excessive or dysregulated activation, it may contribute to cell death [56].

Although autophagy is predominantly a pro-survival mechanism, preventing toxic accumulation and maintaining metabolic balance, it exists at the boundary between survival and death. Excessive or dysregulated autophagy can result in autophagy-related cell death, a regulated form of cell death distinct from apoptosis and necrosis, where uncontrolled self-digestion compromises essential cellular structures. Autophagy also exhibits extensive crosstalk with other death pathways: for example, it can inhibit apoptosis by degrading damaged mitochondria and reducing cytochrome c release, or conversely, promote ferroptosis by influencing iron homeostasis. Following ferritinophagy induction (ferritinophagy is a selective form of autophagy that specifically targets intracellular ferritin), autophagolysosomes degrade ferritin, resulting in the release of free Fe^2+^. This iron fuels the Fenton reaction, amplifying the production of ROS and ferroptosis [57].

### Cell death at the crossroads

For many years, it was generally assumed that, once the process of cell death was activated, it could not be reversed, and the cell was doomed to die. However, it now appears that, despite the molecular activation of a certain type of cell death, some cells can survive this 'near-death experience', which can affect their properties and behaviour. While the extensive MOMP usually seals the fate of stressed cells by triggering the activation of caspases, the partial permeabilisation of the outer mitochondrial membrane (sublethal MOMP) has been associated with tumour cell survival, increased genomic instability, and tumour progression. Sublethal MOMP also triggers a stress adaptation pathway involving cytochrome c, EIF2AK1 and ATF4. This mechanism confers a resistant phenotype to tumour cells that survive sublethal apoptotic signalling, which is associated with increased metastatic spread [58]. The reversibility of certain cell death mechanisms represents a significant window of opportunity for bacterial effects. The microbial signals can shift the balance between survival and death, influencing tumour progression, immune activation, and therapeutic responses. For example, *Chlamydia trachomatis* inhibits apoptosis in infected cells by targeting the pro-apoptotic proteins (BAX, BAK) [59] and p53 degradation [60], creating the possibility of sublethal apoptotic signalling. *Chlamydia trachomatis* also alters the expression of ferroptosis-related proteins and inhibits ferroptosis via the p53-SLC7A11 axis [61].

Cell death is now understood not as a fixed choice between distinct pathways, but as a dynamic network, allowing cells to switch between death programmes depending on context. A key example is the interplay between apoptosis and necroptosis, both regulated by TNF receptors and sharing FADD and caspase-8. Normally, caspase-8 suppresses necroptosis by cleaving RIPK1 and RIPK3, favouring apoptosis. When caspase-8 is inhibited (e.g., by viral infection or mutation), necroptosis proceeds via the RIPK1–RIPK3–MLKL axis, ensuring cell elimination even if apoptosis fails [62]. A clinical example would be infection with *Coxiella burnetii*. *C. burnetii* inhibits TNFα-mediated caspase-8 activation, thereby blocking apoptosis and increasing susceptibility to necroptosis [63]. *Coxiella burnetii* has a demonstrated role in the tumour microenvironment, with its presence associated with an increased risk of B-cell non-Hodgkin lymphoma [64] and the formation of granulomas [65]. In some apoptosis-resistant cancer cells, ubiquitination of RIPK1 via cellular apoptosis inhibitors (cIAPs) or the LUBAC complex can activate NF-κB, promoting survival and inflammation [66]. Indeed, LUBAC determines chemotherapy resistance in squamous cell lung cancer [67]. *Shigella flexneri* suppresses NF-kB nuclear translocation in response to IL-1β and TNF by targeting the LUBAC component HOIP for proteasomal degradation [68]. Some studies showed that *Shigella flexneri* has the potential to induce pro-apoptotic effects in pancreatic cancer cells [69] or macrophages [70].

Pyroptosis and apoptosis are also interconnected. When apoptosis is blocked (e.g., caspase-8 inhibition), caspase-1 can trigger pyroptosis[71, 72]. In GSDMD-deficient cells, caspase-1 induces apoptosis via the BID/caspase-9/caspase-3 axis, sometimes followed by GSDME-mediated pyroptosis. Since GSDME is often silenced in tumours but not healthy tissues, caspase-3 activation may underlie chemotherapy-related toxicities [73, 74]. On the other hand, pyroptotic caspase-1 can cleave BID to activate apoptosis, particularly in cells with little or no GSDMD (e.g., neurons, mast cells). While inflammasomes induce pyroptosis, caspase-1 also activates caspase-3 and 7, which inactivate GSDMD, creating feedback to limit inflammation [14]. This mechanism is co-opted by *Shigella dysenteriae* (specifically serotype 1) and Shiga toxin-producing *Escherichia coli*. Shiga toxin recruits caspase-3 to cleave an active pore-forming N-terminal fragment of GSDMD, thereby preventing the formation of membrane pores required for pyroptosis [75].

PANoptosis is a recently proposed concept in cell death biology that describes a coordinated and interconnected form of cell death, integrating features of pyroptosis, apoptosis, and necroptosis. The term highlights that cell death is not always a linear or isolated process; instead, cells may activate multiple overlapping death programmes depending on the type and intensity of stress. PANoptosis involves simultaneous or sequential activation of apoptotic caspases (e.g., caspase-3, caspase-8), pyroptotic inflammasomes (e.g., caspase-1, gasdermins), and necroptotic mediators (RIPK1, RIPK3, MLKL). It can be induced by microbial infections, inflammatory signals (such as TNF-α or interferons), or genotoxic and metabolic stress [76]. ZBP1 (Z-DNA binding protein 1) is an innate immune receptor that senses nucleic acids in the unusual Z-conformation and triggers PANoptosis. Upon activation, ZBP1 forms a complex with RIPK3 and caspase-8, creating a signalling scaffold that drives NLRP3 inflammasome–dependent pyroptosis, caspase-8–mediated apoptosis, and RIPK3–MLKL necroptosis [77]. Z-DNA has been found in the matrix of *Streptococcus mutans* biofilms and in dental biofilms. *Streptococcus mutans* has been shown to promote the progression of oral squamous cell carcinoma (OSCC) through inflammation and NF-κB activation [78, 79]. Mechanistically, *Streptococcus mutans* infection can promote chronic oral inflammation through persistent activation of innate immune sensors (such as ZBP1). This can trigger PANoptosis and the release of pro-inflammatory cytokines and DAMPs, sustaining a tumour-promoting microenvironment. In OSCC, where apoptosis pathways are often dysregulated [80], PANoptosis-related inflammation may represent an underexplored driver of OSCC progression. Bacteria and LPS strongly enhance inflammatory cell death via the PANoptosis pathway, involving caspase-1, caspase-11, caspase-8, and RIPK3, with caspase-7 also contributing. Additionally, NINJ1 acts as a key executioner mediating the release of inflammatory molecules independently of gasdermin D, gasdermin E, and MLKL [81].

Some proteins and molecules, such as ROS, nicotinamide adenine dinucleotide (NAD⁺), and p53, occupy central positions within the network of interconnected cell death pathways. Modulating their activity can influence multiple forms of regulated cell death. For example, p53 induces apoptosis in response to DNA damage by activating pro-apoptotic genes such as *PUMA*, *BAX*, and *NOXA*. It also acts through transcription-independent mechanisms, binding anti-apoptotic proteins (BCL-2, BCL-XL, MCL-1) or directly activating BAK, thereby promoting intrinsic patway of apoptosis [82]. It can also promote ferroptosis by repressing the cystine/glutamate antiporter (SLC7A11), leading to lipid peroxidation [83, 84]. In addition, p53 contributes to necroptosis via downregulation of sulfiredoxin and peroxiredoxin [85] and can modulate autophagic flux by controlling genes like DRAM1 [86]. p53 also regulates the biogenesis of iron-sulphur clusters and the copper chelator glutathione, key components of the cuproptotic pathway, indicating a potential role for p53 in cuproptosis [87]. Importantly, the type of cell death induced often depends on cellular context, stress intensity, and crosstalk with other signalling pathways. Consequently, microbiome and microbial metabolites can shift the balance between survival and death or types of cell death. The distinct form of cell death not only determines cellular fate but also shapes tissue-level and systemic outcomes. This demonstrates the potential of the microbiome to not only influence tumour progression but also shape therapeutic responses by fine-tuning the network of cell death pathways. For example, gut microbes profoundly influence p53 signalling in colorectal cancer. Pathogenic *E. coli* strains, particularly pks + *E. coli*, contribute to tumorigenesis by producing colibactin, which induces a senescence-associated secretory phenotype [88] and increases p53 mutations [89], thereby promoting cancer progression through the Wnt/β-catenin pathway [90]. Colibactin synthesis by pks + E. coli requires spermidine, a polyamine abundant in cancer tissues and produced by gut microbes. Loss of spermidine synthase reduced colibactin-induced DNA damage, which was restored by spermidine supplementation [91, 92]. OmpA protein was overexpressed in *E. coli* from the B2 phylogenetic group isolated from colorectal cancer (CRC) patients, and it markedly suppressed the expression of the pro-apoptotic genes *BAX* and *BAK* in the HCT116 cells [93]. Conversely, probiotics such as *Lactobacillus rhamnosus* and *L. plantarum* enhance p53 and BAX expression, promoting apoptosis and suppressing tumour growth [94, 95]. Another gut probiotic, *Akkermansia muciniphila,* also upregulates p53 [96]. Together, these findings underscore the role of gut microbes in modulating cell death through p53 pathways.

## NAD⁺– microbiome axis in cell death control

Nicotinamide adenine dinucleotide (NAD⁺) is a pivotal metabolite that connects energy metabolism, DNA repair, and redox balance. Due to its role in the cell's antioxidant defence, NAD⁺ availability directly influences several forms of regulated cell death, positioning it as a central node in determining cell fate under stress conditions [97, 98]. Apoptosis is closely linked to NAD⁺ homeostasis through the activity of the NAD⁺-dependent histone deacetylase SIRT1. By removing acetyl groups from lysine residues, SIRT1 regulates key apoptotic pathways [99]. For instance, deacetylation of p53 (e.g., at C-terminal lysine 382) [100] reduces its ability to activate pro-apoptotic genes such as PUMA, thereby suppressing p53-dependent apoptosis [99, 101]. SIRT1 also modulates BCL-2 family proteins [99], represses FOXO-driven apoptosis under stress [102], and attenuates NF-κB signalling to limit inflammasome activation [103]. Importantly, NAD⁺ precursors such as nicotinic acid and nicotinamide provide cytoprotection against apoptosis induced by oxidative and ER stress, further emphasising the role of NAD⁺ in maintaining cell survival under stress [104]. Beyond apoptosis, SIRTs also regulate autophagy by modulating Atg proteins or AMPK activation, which promotes autophagy and mitigates apoptosis in hypoxic stress [105]. SIRT1 transient upregulation is sufficient to enhance basal autophagy [106]. SIRT1 plays a central role in autophagy regulation by deacetylating Atg proteins (Atg5, Atg7, Atg8) [106] as well as Beclin-1, LC3, and ULK1, which together influence autophagic flux and autophagosome–lysosome fusion. Depending on the context, these actions can either promote or impair autophagy, with SIRT1 loss leading to autophagy dysregulation [99].

Severe DNA damage activates PARP-1, causing rapid depletion of NAD⁺ and ATP and accumulation of PAR, which disrupts glycolysis and energy balance, ultimately leading to cell death. Restoring NAD⁺ can reverse these effects as extracellular NAD⁺ enhances PARP-dependent DNA repair capacity [107]; for example, NAD⁺ supplementation protects mouse astrocytes from PARP-1–induced glycolytic inhibition and cell death [108]. Similarly, in models of septic shock, NAD⁺ administration has been shown to block pyroptosis and improve survival outcomes [109]. Moreover, NAD⁺ treatment prevents rotenone-induced apoptosis and necrosis in pheochromocytoma cells [110]. Under oxidative stress, NAD⁺ dynamics dictate cell death mode. In H_2_O_2_-treated HeLa cells, PARP1 depletes NAD⁺. Under mild stress, nicotinamide phosphoribosyl transferase-dependent salvage restores NAD⁺ and ATP synthesis, supporting ATP-dependent apoptosis. Severe stress impairs NAD⁺ recovery, blocking apoptosis and promoting necrosis, highlighting NAD⁺ metabolism as a key switch between cell death pathways [97]. Beyond individual pathways, NAD⁺ depletion is sensed by NLRC5, which assembles the PANoptosome to trigger PANoptosis while promoting inflammation [111]. These findings highlight NAD⁺ as a central metabolic hub that not only governs energy homeostasis but also orchestrates complex, integrated cell death and inflammatory responses, highlighting its broad role across distinct cell death pathways. NAD⁺ also plays a key role in antitumour immune responses, as regulatory T cells (Tregs; immunosuppressive immune cells that hinder anti-tumour responses and promote cancer progression) are susceptible to NAD⁺-induced cell death [112].

**Fig. 1:**
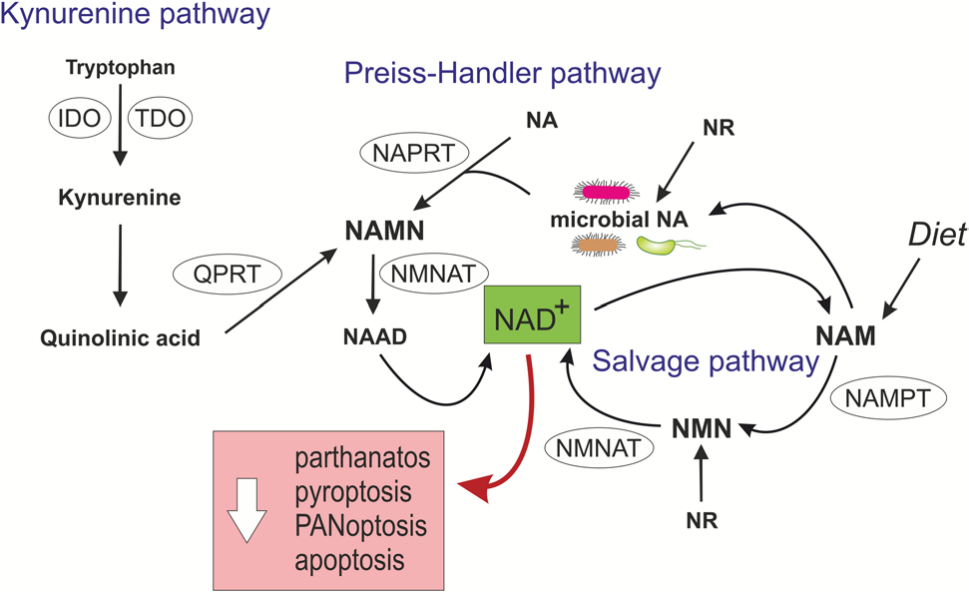
Nicotinamide adenine dinucleotide (NAD⁺) metabolism and its connection with cell death. NAD⁺ is a key metabolite linking energy metabolism, DNA repair, and redox homeostasis. By supporting the cell’s antioxidant defence, NAD⁺ availability critically regulates multiple forms of programmed cell death, making it a central determinant of cell fate under stress. NAD⁺ can be synthesised through three major biochemical routes: the kynurenine pathway, the Preiss–Handler pathway, and the salvage pathway. In the kynurenine pathway, kynurenine is produced from tryptophan, with Tryptophan 2,3-dioxygenase (TDO) and Indoleamine 2,3-dioxygenase (IDO) acting as rate-limiting enzymes. Kynurenine then undergoes several conversions to yield quinolinic acid. Quinolinic phosphoribosyltransferase (QPRT) converts quinolinic acid into nicotinic acid mononucleotide (NAMN), which enters the Preiss–Handler pathway. In the Preiss–Handler pathway, nicotinic acid (NA) is converted to NAMN by nicotinic acid phosphoribosyltransferase (NAPRT). NAMN is then converted to nicotinic acid adenine dinucleotide (NAAD) by nicotinamide mononucleotide adenylyltransferase 1 (NMNAT1), and finally to NAD⁺ by NAD⁺ synthase (NADS). In the salvage pathway, NAD⁺-consuming reactions generate nicotinamide (NAM) as a by-product, which is recycled into NAD⁺. NAM is first converted to nicotinamide mononucleotide (NMN) by nicotinamide phosphoribosyltransferase (NAMPT), the rate-limiting enzyme of this pathway, and NMN is subsequently converted to NAD⁺ by NMNAT1–3. Nicotinamide riboside (NR) can serve as an additional precursor that is phosphorylated by nicotinamide riboside kinase 1 or 2 (NRK1/2) to generate NMN, linking it to the salvage pathway. Host-derived nicotinamide (NAM) enters the gut to support microbial NAD⁺ synthesis, while microbes convert NAM into nicotinic acid (NA), which is absorbed by intestinal tissues and used for NAD⁺ regeneration via the Preiss–Handler pathway. Because NAPRT is not feedback-inhibited like NAMPT, NA supplementation efficiently boosts NAD⁺. Microbial conversion of NAM and NR helps sustain circulating NA and host metabolic flexibility, maintaining normal NA levels even in the absence of dietary precursors

Except for neurons, mammalian cells cannot import NAD⁺ and must instead produce it. NAD⁺ can be produced via two main pathways: *de novo* synthesis and the salvage pathway (Fig. 1) [113]. *De novo* synthesis occurs through either the kynurenine pathway, which builds NAD⁺ from the amino acid tryptophan and the vitamin nicotinic acid (NA, niacin) via the kynurenine pathway with the help of enzymes tryptophan 2,3-dioxygenase (TDO) or indoleamine 2,3-dioxygenase (IDO), or the Preiss-Handler pathway, which transforms NA to NAD⁺ via nicotinic acid phosphoribosyl transferase (NAPRT). The salvage pathway recycles nicotinamide, NA, nicotinamide riboside (NR), and nicotinamide mononucleotide (NAM) back to NAD⁺, with nicotinamide phosphoribosyl transferase (NAMPT) controlling the rate-limiting step [114]. NR supports NAD⁺ mainly via microbial conversion to NA, underscoring the microbiome’s role [115].

Most tissues depend on the salvage pathway for NAD⁺ regeneration, but the Preiss-Handler pathway becomes more important under high NA availability. NAD⁺ precursors are produced both by the host and the gut microbiome, forming a symbiotic cycle in NAD⁺ metabolism. Host-derived nicotinamide (NAM) enters the gut to fuel microbial NAD⁺ synthesis, while microbes convert it into NA that can be taken up by host intestinal tissues and used to regenerate NAD^+^ via the Preiss-Handler pathway. Unlike NAMPT, NAPRT is not feedback-inhibited, making NA supplementation an efficient way to boost NAD⁺. The microbiome-driven transformation of precursors, including NAM and NR, is essential for maintaining circulating NA and supporting the host’s metabolic flexibility; even with a diet free of both NAM and NA, microbial NA production remains high enough to maintain normal levels of circulating NA in the host [115]. Moreover, NA (as well as butyrate [116]) activates the GPR109A receptor on epithelial and immune cells, where it can exert anti-inflammatory and tumour-suppressive effects, including cytokine regulation, immune cell differentiation, and induction of apoptosis. GPR109A is expressed in normal mammary tissue but silenced in breast tumours and cancer cell lines. Restoring its function reduces cAMP, induces apoptosis, and suppresses tumour growth, while GPR109A deletion in mice accelerates mammary tumorigenesis and metastasis [117].

### NAD⁺ in the context of cancer

While ATP is essential for cancer cell function, some research suggests that the demand for NAD⁺ may be even greater than that of ATP in rapidly proliferating cells, as it is crucial for maintaining cellular redox balance to prevent cell death. NAD⁺ regeneration is also essential for *de novo* lipid synthesis. In the absence of exogenous lipids, limited NAD⁺ availability impaired cell proliferation and lipid synthesis, but supplementation with alternative electron acceptors (pyruvate, α-ketobutyrate, or NADH oxidase from *Lactobacillus brevis*) restored proliferation [118, 119]. Li et al. also showed that acetate (as a direct carbon source for cytosolic acetyl-CoA) can bypass the NAD⁺ consuming reactions involved in *de novo* lipid synthesis [119]. The increased NAD⁺ requirement may explain why cancer cells rely heavily on lactate fermentation. Lactate fermentation regenerates NAD⁺ by converting NADH (produced during glycolysis) back to NAD⁺ as pyruvate is converted to lactate (reaction catalysed by the enzyme lactate dehydrogenase). Normally, cells produce NAD⁺ and ATP together through respiration. However, if a cell has excess ATP, it can slow down the entire process, including NAD⁺ production. By increasing lactate production, a cell can bypass this bottleneck, generate more NAD⁺, and thus support rapid proliferation, even if it's less efficient in ATP production [120]. Consequently, cancer cells often exhibit a metabolic phenotype known as the Warburg effect, characterised by high rates of glycolysis and lactate production even in the presence of oxygen. Moreover, mitochondria-bound hexokinase, a key glycolytic enzyme, promotes apoptosis resistance, suggesting that glycolysis may protect tumour cells from apoptosis [121]. In line with this, Zheng et al. showed that *Fusobacterium nucleatum* infection elevates ANGPTL4 expression, which enhances glucose uptake and glycolysis in colorectal cancer cells, thereby supporting both bacterial colonisation and tumour survival [122]. Moreover, *F. nucleatum* outer membrane proteins Fap2 and RadD induce cell death in human T-lymphocytes [123].

In replenishing the intracellular NAD⁺ pool, NAMPT also plays a critical role. Accordingly, the NAMPT enzyme is overexpressed in many cancers, including glioma, lymphoma, breast, prostate, gastric cancer, and bladder cancer, typically linked to poor patient prognosis, advanced disease stage, aggressive tumour behaviour, and chemotherapy resistance [124, 125]. NAMPT can also induce stemness in glioma cells [126]. NAMPT is highly expressed in immunosuppressive tumour-associated macrophages (TAMs) and supports tumour immune evasion. Importantly, tumours with high NAMPT expression are more responsive to anti-PD-L1 therapy, as NAMPT drives PD-L1 expression via NAD⁺-dependent TET1 and STAT1/IRF1 signalling [127].

Blocking NAMPT with NAMPT inhibitors depletes NAD⁺, impairing vital processes, which leads to cancer cell death and attenuates tumour growth. NAMPT inhibition triggers apoptosis in acute myeloid leukaemia stem cells by disrupting lipid homeostasis while sparing normal hematopoietic stem cells [128]. Although early studies of NAMT inhibitors showed promise, clinical development has been limited by dose-related toxicities (especially thrombocytopenia) and modest effectiveness as standalone therapies [125]. Interestingly, the microbiome may be the cause of the low efficacy of NAMP inhibitors. Microbiome-mediated resistance to NAMPT inhibitors arises because gut bacteria possess a nicotinamidase (PncA) enzyme that converts NAM into NA. As a key substrate of the Preiss–Handler pathway via NAPRT, NA supports NAD⁺ synthesis. Consequently, this bacterial action bypasses the inhibited NAMPT enzyme in the host, especially if certain tumours amplify the *NAPRT* gene [129]. A diet high in nicotinamide precursors can strengthen this resistance, while antibiotics that reduce gut microbiota can potentially overcome it by eliminating the bacteria responsible for this NAD⁺ salvaging mechanism [130, 131]. *Mycoplasma hyorhinis*, a frequent contaminant of tumour tissues, has been shown to express PncA, thereby redirecting NAD synthesis from the NAMPT-dependent salvage pathway toward the Preiss–Handler pathway, bypassing pharmacological inhibition of NAMPT, and thus conferring resistance to NAMPT inhibitors. Beyond resistance to NAMPT inhibitors, the microbial rerouting of NAD precursors may enhance tumour cell adaptability to ploidy changes [132] and influence immune responses within the TME. Chronic *M. hyorhinis* infection activates NF-κB signalling, driving pro-inflammatory cytokine and chemokine expression, and enhancing proliferation, migration, and invasion of cancer cells [133, 134]. These findings underscore the need to consider microbial contributions, such as NAD rewiring, when designing and evaluating NAD-targeted therapies in cancer.

## Tryptophan metabolites and cell death control

Dynamic interaction between host and microbiome makes tryptophan (Trp) metabolism a critical factor in shaping cell death in the TME. Trp is an essential dietary amino acid, serving not only in protein synthesis but also as a precursor for key metabolites. Trp is processed by both host and microbial enzymes. Cancer cells often deplete Trp through high activity of indoleamine 2,3-dioxygenase (IDO) and tryptophan 2,3-dioxygenase (TDO), diverting it into the kynurenine pathway to support immune evasion. The hyperactivity of the kynurenine pathway elevates the kynurenine/tryptophan ratio, a marker of immune suppression and poor prognosis, as high kynurenine levels promote tumour immune evasion and lead to T-cell apoptosis [135]. Moreover, Trp deprivation halted cell growth and led to cell death in human pluripotent stem cells [136]. In mouse models of lung cancer, Trp supplementation or IDO inhibition enhanced CD8⁺ T-cell–mediated apoptosis of cancer cells, increased CD8⁺ T-cell infiltration into tumours, and slowed tumour growth. Trp potentiated anticancer activity of CD8^+^ T-cells by TRIP12 tryptophanylation and CD8^+^ T-cell surface PD-1 downregulation [137].

The shortage of Trp not only suppresses T-cell proliferation but also reshapes the microbiome. Many commensal gut bacteria are Trp auxotrophs that depend on environmental supply. For instance, the *Ruminococcaceae* species *Faecalibacterium prausnitzii* or *Subdoligranulum variabile* [138] *or Actinobacteria*, such as *Collinsella* and *Olsenella* [139], require external Trp to grow and to produce butyrate [140] that normally strengthens epithelial barrier function and regulates mucosal immunity [141, 142]. Patients who responded well to immunotherapy had higher levels of *Ruminococcaceae* and *Collinsella* in their gut, which correlated with stronger and longer-lasting treatment effects and increased T-cell infiltration in tumours [143, 144]. In a Trp-depleted TME, these beneficial bacteria may decline.

**Fig. 2:**
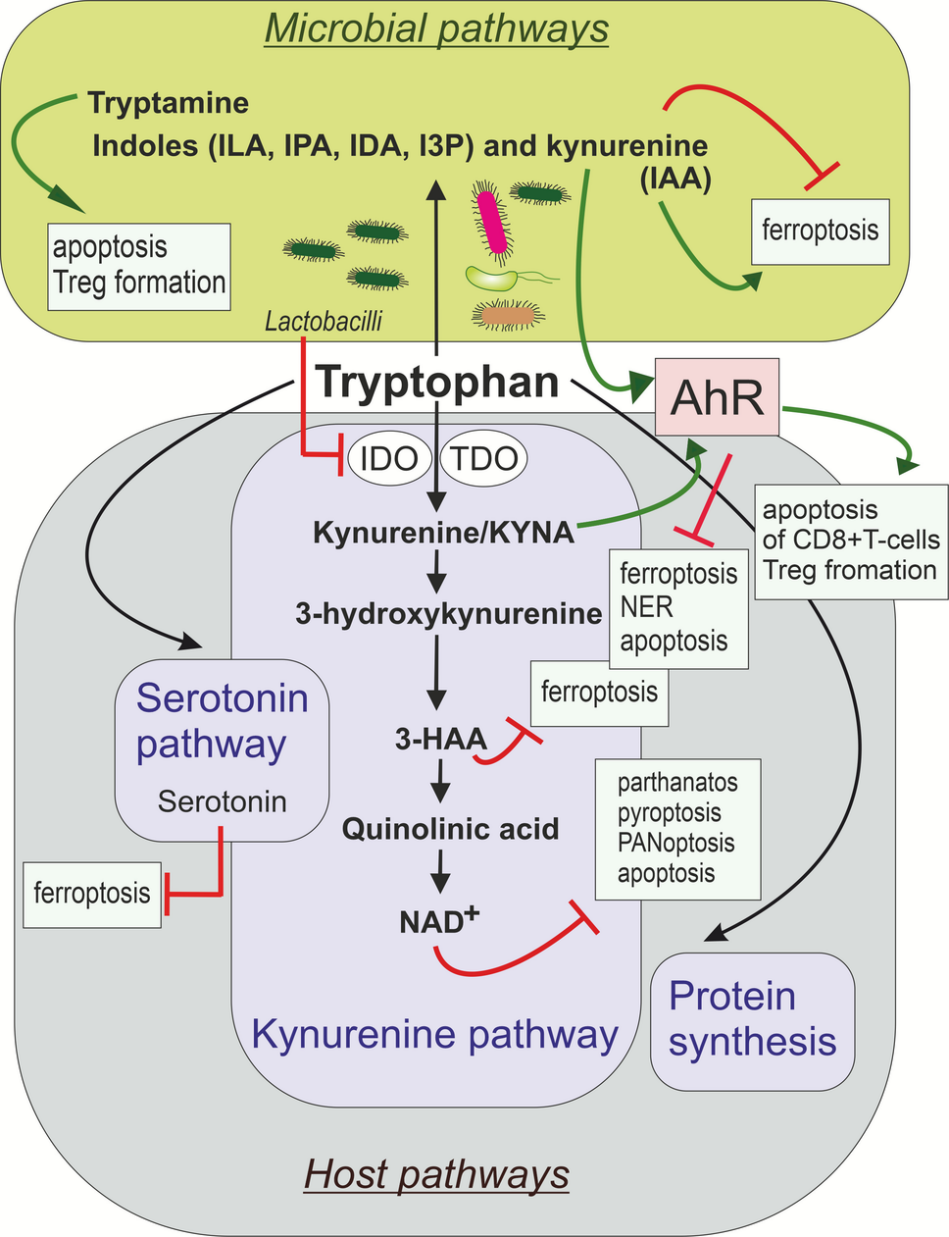
Tryptophan metabolism and its connection with cell death. In host cells, tryptophan is metabolised through the kynurenine and serotonin pathways to generate bioactive metabolites or incorporated directly into protein synthesis. Cancer cells often deplete tryptophan through high activity of indoleamine 2,3-dioxygenase (IDO) and tryptophan 2,3-dioxygenase (TDO), diverting it into the kynurenine pathway to support immune evasion. Lactobacilli produce hydrogen peroxide, which suppresses IDO and the host kynurenine production. A branch of the kynurenine pathway is involved in *de novo* NAD⁺ biosynthesis. Due to its role in the cell's antioxidant defence, NAD⁺ availability directly influences several forms of regulated cell death. In gut microbes, tryptophan is transformed into kynurenine, indoles, and derivatives such as tryptamine. These metabolites support intestinal barrier integrity and immune balance by activating the aryl hydrocarbon receptor (AhR), which promotes apoptosis of CD8^+^T-cells, generation of regulatory T-cell (Treg), and an immunotolerant microenvironment. In the tumour microenvironment, active AhR can promote immune evasion and drive cancerogenesis via repressing nucleotide excision repair (NER), apoptosis, and ferroptosis. Serotonin, 3-hydroxyanthranilic acid (3-HAA), indole‐3‐lactic acid (ILA), indole-3-propionic acid (IPA), trans-3-indoleacrylic acid (IDA), indole-3-pyruvate (I3P), and kynurenine were shown to inhibit ferroptosis. In contrast, indole-3-acetic acid (IAA) can promote ferroptosis

In the host, Trp can be used for protein synthesis (~ 1%) or can be metabolised through the kynurenine (~ 95%) (Fig. 2) or serotonin (~ 2%) pathways [145]. The microbiome adds another layer of complexity. The microbiota-dependent “pseudoendogenous” pathway converts Trp into a range of indole derivatives and other bioactive metabolites. Many gut bacteria, such as *E. coli* and *Clostridium* species, metabolise Trp into ligands for the aryl hydrocarbon receptor (AhR) and modulate mucosal immunity, gut barrier integrity, and inflammation [146]. *Lactobacilli* (such as *Lactobacillus johnsonii*) further shape this process by producing hydrogen peroxide, which suppresses host kynurenine metabolism through inhibition of IDO, the enzyme that catalyses kynurenine biosynthesis from Trp [147]. In cancer, kynurenine contributes to immune evasion and therapy resistance by signalling via AhR. Moreover, kynurenine can inhibit ferroptosis. Inside receiver cells, kynurenine activates an NRF2-dependent, AhR-independent pathway that upregulates expression of SLC7A11 (protein crucial for preventing ferroptosis, also known as xCT), thereby promoting further kynurenine import [148]. Kynurenine can also induce the expression of PD-1 in  CD8^+^T-cells [149] and simultaneously can increase PD-L1 expression on tumour cells [150]. The binding of PD-L1 to PD-1 can lead to the apoptosis of activated T-cells, reducing the anti-tumour immune response [151]. Consequently, the anticancer effects of *L. johnsonii* can be mediated through the modulation of kynurenine in tumour cells. In papillary thyroid carcinoma (PTC), patients with lymph node metastasis show a significant reduction of *L. johnsonii* in tumour lesions. Gut colonisation with *L. johnsonii* increases its abundance in tumours, leading to inhibition of PTC growth and metastasis [152]. Conversely*, Streptococcus mutans* colonises OSCC tumours and drives excessive kynurenic acid (KYNA) production through its protein antigen c (PAc). KYNA promotes expansion of IL-1β–producing neutrophils, leading to CD8^+^T-cell exhaustion and apoptosis, immunosuppression, and OSCC progression. It also reduces the efficacy of PD-L1 and IL-1β blockade therapies, with high KYNA–AhR signalling correlating with poor patient survival [153].

Dietary fibre serves as an important determinant of gut microbial Trp metabolism. In the gut, *E. coli* converts Trp into indole, while *Clostridium sporogenes* uses it in the Stickland pathway to regenerate NAD⁺ and produce indole‐3‐lactic acid (ILA) and indole-3-propionic acid (IPA). The fibre degrader *Bacteroides thetaiotaomicron* (indole producer, pectin degrader) breaks down pectin into monosaccharides that repress *E. coli*’s *tnaA* gene (tryptophanase), leaving more Trp available for Stickland fermenters [154]. ILA was shown to inhibit tumour cell proliferation, migration, and anti-apoptotic capabilities [155]. ILA also inhibits ferroptosis through activating the AhR/Nrf2 signalling pathway [156]. *Lactobacillus johnsonii* collaborates with *Clostridium sporogenes* to produce IPA. IPA modulates the stemness programme of CD8 + T-cells and improves responsiveness to immune checkpoint blockade at the pan-cancer level, including melanoma, breast cancer, and colorectal cancer [157]. IPA supplementation can inhibit ferroptosis and oxidative stress through the NRF2/GPX4 axis [158]. IPA also induces Ca^2+^-dependent apoptosis in *Candida albicans *[159]. *Candida albicans* acts as a driver of chronic inflammation, metabolic competition, and immune suppression in the TME, supporting tumour progression and potentially reducing therapeutic efficacy [160]. Several other indole derivatives produced by bacteria have been shown to act as potent ferroptosis inhibitors. 3-hydroxyindole, 6-hydroxyindole, 7-hydroxyindole, and indole-3-pyruvic acid suppress ferroptosis by scavenging free radicals and enhancing antioxidant gene expression [161, 162]. Trans-3-indoleacrylic acid (IDA), produced by *Peptostreptococcus anaerobius*, inhibits ferroptosis in tumour cells through a mechanism independent of lipid peroxidation. IDA treatment or implantation of *P. anaerobius* promotes colorectal cancer progression in both the xenograft model and ApcMin/+ mice via IDA–AhR–ALDH1A3 axis [163]. Additionally, indole and 7-hydroxyindole can reduce *Pseudomonas aeruginosa* virulence [164]. In contrast, *Parabacteroides distasonis*-derived indole-3-acetic acid (IAA) can promote ferroptosis by reducing FASN expression, decreasing the ratio of MUFAs to PUFAs, thereby increasing ferroptosis sensitivity in bladder cancer cells. *P. distasonis* is significantly decreased in bladder cancer patients, and its low abundance is strongly associated with poor prognosis [165]. Indoxyl sulphate has been shown to trigger apoptosis through mitochondrial ROS production [166, 167]. Indole-3-carboxaldehyde (ICA) is a gut microbe-derived tryptophan metabolite. ICA acts as a signalling molecule in the gut-brain axis, potentially improving stress and depression-like behaviours by influencing the AhR pathway. Supplementation with ICA or the ICA-producing strain *Lactobacillus reuteri* reduced neuroinflammation and promoted hippocampal neurogenesis via the AhR/SOCS2/NF-κB/NLRP3 pathway, alleviating pyroptosis [168]. Non-Trp-derived indole compounds that have a significant effect on cell death and inflammation include indole-3-carbinol (I3C), which originates primarily from the breakdown of glucosinolates in cruciferous vegetables, and its metabolite 3,3′-diindolylmethane (DIM). These metabolites induce apoptosis in cervical cancer cells by reducing BCL-2 expression [169] and trigger apoptosis in gastric adenocarcinoma cells via the mitochondrial pathway [170]. I3C can also reduce colitis by promoting the production of anti-inflammatory compounds like butyrate and modulating the gut microbiota [171].

Another active metabolites of Trp are serotonin and 3-hydroxyanthranilic acid (3-HAA). Serotonin and 3-HAA protect tumour cells from ferroptosis by acting as radical-trapping antioxidants that block lipid peroxidation. Monoamine oxidase degrades serotonin and abolishes its protective effect, while kynureninase promotes ferroptosis resistance via 3-HAA production. Conversely, 3-hydroxyanthranilate 3,4-dioxygenase consumes 3-HAA, restoring ferroptosis sensitivity, with its expression correlating with higher lipid peroxidation of cancer cells and better clinical outcomes of cancer patients [172]. On the other hand, 3-HAA suppresses NF-κB activation after T-cell receptor signalling by targeting PDK1, leading to apoptosis of activated Th2 cells [173] and monocyte-derived cells stimulated by interferon-gamma [174]. Th2 cells can suppress the growth of colon and pancreatic cancer by activating anti-tumour macrophage and eosinophil responses [175].

At least one bacterium species (such as *Ruminococcus gnavus* and *Clostridium sporogenes*) that encodes a tryptophan decarboxylase can be found in the gut microbiome of > 10% of humans. Such bacteria can produce tryptamine via tryptophan decarboxylation [176]. Tryptamine shows a dual role in cancer. *In vitro*, it inhibited proliferation, induced apoptosis, and reduced migration of prostate cancer cells (PC-3, LNCaP), with weaker effects on normal prostate cells (RWPE-1). *In vivo*, intratumorally injected tryptamine suppressed PC-3 xenograft growth, but systemic (i.p.) administration paradoxically promoted tumour progression [177]. This duality may be linked to its strong immunomodulatory activity: in an EAE mouse model, tryptamine reduced CD4⁺ and Th17 infiltration while expanding FoxP3⁺ regulatory T-cells (Tregs). Because Tregs suppress anti-tumour immunity and support tumour progression, tryptamine’s effects likely depend on the balance between its direct tumouricidal activity and its capacity to promote immune evasion [178]. Tryptamine was also shown to induce cell death with ultrastructural features of autophagy in neurons and glia [179]. The oral commensal *Corynebacterium durum* can convert tryptamine into an anticancer metabolite, durumamide A, an oxidative dimer of tryptamine. Durumamide A showed cytotoxicity across cancer cell lines (IC_50_: 25–35 μM) by targeting surviving (BIRC5) and triggering AIF-mediated apoptosis [180].

## Short-chain fatty acids and bile acids

Short-chain fatty acids (SCFAs), including butyrate, acetate, and propionate, are produced by anaerobic bacteria through the fermentation of dietary fibres in the gut. Beyond serving as an energy source for colonocytes, SCFAs play a critical role in shaping the TME and regulating cancer cell fate by linking microbial metabolism of dietary fibre to both direct cancer cell death pathways and the modulation of anti-tumour immunity [181]. In the gut, *Bacteroidetes* commonly produce acetate, while propionate is generated by species such as *Akkermansia muciniphila*, *Bacteroides vulgatus*, and *B. thetaiotaomicron*. Butyrate is synthesised via acetyl-CoA, glutamate, lysine, or succinate pathways, mainly by clostridial clusters XIVa and IV, which include abundant species like *Faecalibacterium prausnitzii *[116]. Butyrate, in particular, functions as a histone deacetylase (HDAC) inhibitor, promoting apoptosis and cell-cycle arrest in cancer cells while sparing normal cells through the so-called “butyrate paradox.” The metabolic switch to aerobic glycolysis, known as the “Warburg effect,” limits cancer cells’ ability to oxidise butyrate. As a result, butyrate accumulates intracellularly, acting as an HDAC inhibitor and suppressing proliferation [182]. HDAC inhibitors butyrate and suberoylanilide hydroxamic acid (SAHA; vorinostat) trigger caspase-3 activation via the mitochondrial apoptotic pathway but can also induce caspase-independent autophagic cell death in multiple human cancer cell lines. This dual mechanism highlights their potential for treating cancers resistant to apoptosis [183]. Via HDAC inhibition, butyrate also potentiates lipoteichoic acid (LTA)-induced inflammasome activation [184]. Examples of bacteria that produce LTA include species of *Staphylococcus*, *Streptococcus*, and *Bacillus*, as well as many other Gram-positive microorganisms, such as *Enterococcus faecalis *[184]. LTA is a major component of their cell walls [185]. Butyrate and propionate also act as microbial danger signals, activating the NLRP3 inflammasome in human macrophages upon TLR stimulation [186]. Notably, butyrate-producing microbes, such as *Butyricicoccus pullicaecorum*, may counteract the oncogenic effects of p53 mutations in CRC by modulating chromosome segregation 1 (CSE1L) expression [187]. CSE1L is highly expressed in different cancers, correlating with advanced stage, poor prognosis, and worse patient outcomes. It also regulates chemotherapy-induced apoptosis, influencing cancer cell sensitivity to treatment [188, 189]. This suggests a potential microbiome-driven mechanism for restoring tumour-suppressive pathways and slowing cancer progression [190]. SCFAs can also influence other forms of regulated cell death: they modulate ferroptosis by affecting lipid metabolism. Sodium butyrate (NaB) enhances RSL3- and erastin-induced ferroptosis by increasing lipid peroxidation through downregulation of SLC7A11 and GPX4. This occurs via the FFAR2-AKT-NRF2 and FFAR2-mTORC1 axes in a cAMP-PKA-dependent manner. Functionally, NaB suppresses tumour growth, which can be reversed by mTORC1 activation or ferroptosis inhibition, highlighting its potential for colorectal cancer therapy through mTOR-dependent ferroptosis regulation [191]. Butyrate can also activate AMPK [192], which stabilises p53 protein by phosphorylating MDMX and inhibiting its activity [193]. Furthermore, AMPK senses cellular energy status and, when activated, phosphorylates and inhibits RIPK1 to suppress cell death and inflammation under nutrient stress. Nevertheless, prolonged stress overrides this inhibition, leading to cell death [194, 195]. GPR109A, the niacin/butyrate receptor, suppresses mammary tumorigenesis by inhibiting antiapoptotic signalling. Loss of GPR109A increases tumour incidence and lung metastasis in mice [196]. Accordingly, *Lactiplantibacillus plantarum* inhibited colon cancer cell proliferation as a function of its butyrogenic activity [197]. Butyrate and propionate were shown to induce apoptosis in lung and colorectal cancer cells [198, 199]. Moreover, SCFAs affect immune responses, with butyrate enhancing CD8⁺ T-cell function and regulatory T-cell differentiation, thereby indirectly shaping cancer cell survival or elimination [200].

Most circulating acetate in human plasma (50–200 μM) originates from gut microbial metabolism through the fermentation of dietary fibre. Indigestible polysaccharides are degraded by bacterial enzymes into hexoses (glucose, fructose), which are metabolised via glycolysis to pyruvate and then acetyl-CoA. This is converted to acetate through acetyl-phosphate and acetate kinase, generating ATP, or via the Wood–Ljungdahl pathway. There are metabolic links between different types of bacteria; for example, acetate produced by *Bacteroidetes* species can be utilised by *Firmicutes* to produce butyrate [116]. Interestingly, the *Firmicutes*/*Bacteroidetes* ratio was lower in patients with breast or colon cancer than in healthy controls [201, 202]. Non-consumed acetate is then released into the gut lumen. Tumour cells can absorb external acetate either through passive diffusion of acetic acid at low pH or active transport by monocarboxylate transporters (MCTs). Intracellular acetate is rapidly converted to acetyl-CoA by ACSS2 in the cytosol or ACSS1 in mitochondria. Cytosolic acetyl-CoA fuels biosynthesis of fatty acids, isoprenoids, sterols, ketones, and N-acetyl-glucosamines, which support cancer growth and the synthesis of cellular components, or serves as a substrate for lysine acetyltransferases to acetylate metabolites and proteins, including histones [118, 203]. For example, acetate induces expression of lipogenic genes *ACACA* and *FASN* by increasing H3K9, H3K27, and H3K56 acetylation, which enhances *de novo* lipid synthesis. This effect is reflected in hepatocellular carcinoma with high ACSS1/2 expression, where increased histone acetylation and lipid synthesis support cancer cell survival under hypoxia [204, 205]. Under glucose deprivation, AMPK phosphorylates ACSS2 at S659, driving nuclear translocation. In the nucleus, ACSS2 partners with TFEB, strongly promoting lysosomal biogenesis and autophagy, which are critical for tumour cell survival [206]. Acetate can also inhibit pyroptosis by attenuating inflammasome activation via GPR43-mediated Ca^2+^-dependent NLRP3 ubiquitination [207, 208]. On the other hand, acetate (as well as propionate, butyrate, formate, and pentanoate) can activate free fatty acid receptor 2 (FFAR2) and induce apoptosis through ERK signalling [209]. However, cancer cells can escape ERK-induced cell death by remodelling the ERK signalling. For example, pERK1/2 phosphorylates caspase-8 at S387, blocking its activation and thereby protecting cancer cells from caspase-8–mediated apoptosis [210].

The intestinal microbiome is also crucial for bile acid (BA) metabolism, converting primary to secondary BAs via microbial 7α-dehydroxylase and bile salt hydrolase (BSH) enzymes. BSH activity is widespread, especially in gram-positive bacteria such as *Clostridium*, *Enterococcus*, *Bifidobacterium*, *Listeria*, and *Lactobacillus* (e.g., *L. plantarum*). Deoxycholic acid (DCA), a secondary bile acid, influences host metabolism and may drive anti-apoptotic mechanisms by activating pathways such as EGFR (EGFR directly phosphorylates TNFR1 at Tyr360 and Tyr401, modulating NF-κB signalling and TNF-α–induced apoptosis and necroptosis) [211, 212], RelA [213], and Wnt5a/β-catenin [146, 214]. On the other hand, DCA can also trigger cell death by inducing oxidative stress, DNA damage, and mitochondrial dysfunction [215].

## Krebs cycle metabolites lactate and ammonia

Excess NADH, which arises during hypoxia, mitochondrial dysfunction, or Warburg-like metabolic reprogramming, common in cancer, slows the electron transport chain (ETC) and inhibits succinate dehydrogenase (SDH, ETC complex II). This leads to the accumulation of both succinate and fumarate, which act as oncometabolites. Succinate stabilises hypoxia-inducible factor 1α (HIF-1α) by inhibiting prolyl hydroxylases and can also act extracellularly through the SUCNR1 receptor to promote inflammation and angiogenesis. Fumarate, in contrast, strongly modifies proteins through succination (e.g., of KEAP1), leading to NRF2 pathway activation and antioxidant responses. Succination also inhibits proteins such as the tumour suppressor PTEN and gasdermin D (GSDMD), with the latter suppressing pyroptotic cell death [216]. Both endogenous fumarate and exogenous dimethyl fumarate (DMF) succinate cysteine residues in GSDMD, preventing its interaction with caspases and subsequent activation [217]. In the absence of active GSDMD, caspase-1 can trigger apoptosis through the BID/caspase-9/caspase-3 pathway, occasionally followed by GSDME-mediated pyroptosis. Because GSDME is often silenced in tumours [73, 74], succinate may exert greater cytotoxicity in normal cells than in cancer cells. Indeed, succinate triggers pyroptosis in human umbilical vein endothelial cells (HUVECs) via inhibition of ATP5F1D [218], yet ATP5F1D inhibition does not induce pyroptosis in endometrial cancer cells [219]. Furthermore, succinate induces Ufsp2 transcription, promoting high-glucose–triggered pyroptosis in rat retinal Müller cells via NLRP3 inflammasome activation [220]. *UFSP2* is frequently deleted in several human cancers (14 cancer types), and its absence promotes the growth of colon cancer cells and tumours [221]. Succinate also inhibits EGLN3, a key mediator of c-Jun–driven apoptosis [222]. EGLN3 suppresses gastric cancer malignancy by inhibiting JMJD8/NF-κB signalling [223]. Fumarate and succinate also exert epigenetic effects by inhibiting α-ketoglutarate–dependent dioxygenases, including histone and DNA demethylases. This inhibition causes a hypermethylated phenotype, altering the expression of genes controlling DNA repair, apoptosis, cell cycle, differentiation, and adhesion [216]. Together, succinate and fumarate integrate metabolic stress from NADH overload into pseudohypoxia, epigenetic remodelling, and survival signalling, driving tumour progression.

Gut microbes such as *Bacteroides fragilis*, *Ruminococcus albus*, *Faecalibacterium prausnitzii*, and *Akkermansia muciniphila* produce succinate through fermentation pathways. While some succinate is consumed by other gut bacteria, a portion is exported into the circulation, where it acts as a signalling metabolite. Circulating succinate can enter cancer cells through SLC13A3, a transporter directly regulated by β-catenin [224]. A multi-cohort metagenomic analysis of faecal DNA identified a gut bacterial signature—the ratio of succinate producers (*Prevotellaceae* + *Veillonellaceae*) to consumers (*Odoribacteraceae* + *Clostridaceae*)—as a key determinant of plasma succinate, which was higher in obese individuals. Changes in gut microbiota, whether via dietary weight loss or natural shifts, altered circulating succinate levels linked to microbial succinate metabolism [225]. Notably, *Akkermansia muciniphila*, a succinate producer from mucin degradation, is inversely associated with obesity-related metabolic disturbances and is enriched by prebiotics, metformin, and bariatric surgery [226]. This paradox can be partially explained by the trophic interaction between *Akkermansia muciniphila* and the butyrogenic gut commensal *Anaerostipes caccae*. Mucin degradation by *A. muciniphila* promotes *A. caccae* growth and butyrate production via the acetyl-CoA pathway. In turn, *A. caccae* alters *A. muciniphila*’s transcription, upregulating mucin degradation genes and downregulating ribosomal genes [227]. *Clostridiales*, such as *A. caccae*, are linked to lower colorectal tumour burden and are reduced in CRC patients. Oral administration of four Clostridiales strains (CC4) prevented and treated CRC in mice via CD8⁺ T-cell activation, with single strains such as *Anaerostipes caccae* or *Roseburia intestinalis* being even more effective. CC4 supplementation outperformed anti-PD-1 therapy, highlighting gut bacteria (particularly *A. caccae*) as a promising stand-alone cancer therapy [228]. Enterohemorrhagic *E. coli* (EHEC) is linked to CRC through its ability to induce DNA damage, inflammation, and chronic oxidative stress in the colon, which are known contributors to cancer development. EHEC can induce both apoptosis and autophagy in host cells, contributing to intestinal damage [229, 230]. Fumarate significantly increased EHEC virulence [231].

In cancer, high glycolytic flux converts most pyruvate into lactate rather than feeding it into the Krebs cycle (Warburg effect). This rerouting preserves mitochondrial intermediates for biosynthesis while regenerating NAD⁺ to sustain glycolysis under hypoxic or nutrient-stressed conditions. Consequently, tumours often exhibit high lactate levels. Lactate acts as both a metabolic fuel and a signalling molecule that fosters cancer cell survival and therapy resistance [232]. Some Lactate-producing bacteria, such as *Lactobacillus iners* and *Staphylococcus*, found within tumours can even enhance lactate production and contribute to cancer progression, rewiring tumour cell metabolism to fuel growth and resistance to cell death during treatments like chemotherapy and radiation [233, 234]. Lactate accumulation in tumours not only acidifies the microenvironment but also prevents glucose deprivation-induced parthanatos in gastric cancer cells [235] or melanoma cells [236] and suppresses apoptosis by stabilising HIF-1α via lysine lactylation at species-specific residues (K644 in mice and K12 in humans), enhancing protein stability by preventing VHL-mediated recognition [237]. HIF-1α promotes resistance to cell death via drug efflux pumps and accelerated DNA repair [238] and drives cancer resistance to cuproptosis [239]. Mitochondrial HIF-1α also protects against oxidative stress-induced apoptosis by reducing ROS levels and mitochondrial damage [240].

In healthy adults, lactate rarely accumulates in the colon, as it is consumed by other bacteria, including propionate- and butyrate-producers. Lactate, however, is a major fermentation product in breast-fed infants with *Bifidobacterium*-dominated microbiota. In adults, lactate accumulation is linked to dysbiosis, such as in severe colitis, often due to a deficiency of lactate-utilising bacteria [241]. These microbes, including species such as *Anaerostipes caccae*, *Anaerobutyricum soehngenii* (formerly *Eubacterium hallii*), and *Veillonella parvula*, convert lactate into metabolites like butyrate, propionate, and succinate [242]. In the context of the TME, *Veillonella parvula* can inhibit breast tumour growth and metastasis by selectively colonising tumours and reducing intratumoral lactate levels [243]. Proliferation-inhibiting, invasion-inhibiting, and apoptosis-promoting function of *Veillonella parvula* was also observed in oral squamous cell carcinoma [244]. Engineered *E. hallii* is being explored for use as a living biomaterial to convert intratumoral lactic acid into butyrate [245].

In addition to anaerobic glycolysis, cancer cells undergo metabolic reprogramming that heightens their reliance on glutamine. Glutamine supplies energy and carbon/nitrogen for the synthesis of amino acids, nucleotides, and fatty acids. The gut bacteria were shown to reshape amino acid metabolism. Compared to germ-free mice, conventional mice show higher portal vein glutamine but lower levels of many other amino acids (Arg, Asn, His, Ile, Leu, Met, Phe, Pro, Ser, Thr, Trp, Tyr, Val), indicating that microbes boost systemic glutamine while limiting the level of other amino acids [246]. Under hypoxia, glutamine fuels lipid synthesis via reductive carboxylation. This glutamine flux into α-ketoglutarate releases ammonia, leading to high ammonia levels (1–10 mM) in the TME, despite partial reuse by cancer cells [247]. Ammonia accumulation in the TME can become toxic to cancer cells when it exceeds buffering or recycling capacity. Excess ammonia raises lysosomal pH, disrupting lysosomal storage and causing ammonia reflux into mitochondria. This leads to mitochondrial damage and cell death, marked by lysosomal alkalization, mitochondrial swelling, and impaired autophagic flux [248]. Nevertheless, acidification of the TME by lactate can have a profound effect on ammonia toxicity. Mechanistically, increased glutaminolysis produces excess NH4⁺, which is normally exported from mitochondria and cleared from the microenvironment by proper perfusion. When perfusion is impaired, ammonia accumulates outside the cell. At physiological pH, ~ 2.2% of total ammonia exists as NH3, which can freely diffuse across the plasma membrane into the acidic cytosol. If NH3 influx surpasses NH4⁺ efflux, ammonia builds up inside the cell, alkalinizing acidic organelles, disrupting their function, and impairing cell growth and survival. At lower extracellular pH (6.8), only ~ 0.4% of ammonia exists as NH3, and the reversed pH gradient limits its entry into the cytosol. As a result, high extracellular ammonia has little effect under acidic conditions [247]. Consequently, high blood ammonia can be lethal to normal cells, such as CD8^+^ T-cells [249], but tumour cells are shielded by lactate-induced acidosis within the TME. CRC patients exhibit elevated serum ammonia and an ammonia-related gene signature, which is associated with T-cell apoptosis, weakened immune responses, poor prognosis, and resistance to immune checkpoint therapy [249]. The microbial involvement should be considered when assessing ammonia production and its systemic effects, as ammonia is the main nitrogen source in the gut, where many microbes both produce and utilise it, thereby influencing blood ammonia levels. Some microbes generate ammonia either by urease-mediated urea breakdown (e.g., *Enterobacteriaceae,* such as *Klebsiellae*, *Veillonellaceae*, *Staphylococcus aureus*, *Mycobacterium tuberculosis,* or *H. pylori*) or by proteolysis (e.g., *Bacteroides*, *Clostridia*, *Fusobacterium*) [250, 251]. On the other hand, some bacteria also consume ammonia, supporting detoxification. For example, a genetically engineered *Lactobacillus plantarum* strain efficiently converted ammonia into alanine, markedly lowering ammonia levels in both blood and feces [252]. Bell et al. have shown that promoting ammonia clearance attenuates apoptosis in T-cells and reactivates them, reduces tumour growth, and prolongs survival in CRC patients. Lowering ammonia also improves anti-PD-L1 efficacy, suggesting that targeting ammonia detoxification is a promising strategy to boost immunotherapy [249].

## Polyamines, formate, and other bacterial metabolites

Polyamines, including putrescine, spermidine, and spermine, are small, positively charged metabolites produced by both host cells and the gut microbiota. Microbial polyamines boost colonic epithelial proliferation and shape macrophage differentiation. Colonisation of germ-free mice with wild-type, but not polyamine biosynthesis-deficient *Escherichia coli*, elevated intracellular polyamines in colonocytes, accelerating epithelial renewal. Additionally, bacterially derived putrescine promotes anti-inflammatory macrophages in the colon [253]. Polyamines are indispensable for cellular growth and survival, as they stabilise DNA and RNA, regulate ion channels, and support protein synthesis and cell cycle progression. When polyamine synthesis is reduced by mutations or pharmacological inhibition, cancer cells undergo senescence and apoptosis. Polyamine metabolism is tightly linked to the urea cycle through the intermediate ornithine, generated when arginase hydrolyses arginine. The primary polyamine produced from the urea cycle is putrescine, which is formed from ornithine by the enzyme ornithine decarboxylase (ODC). Putrescine is then further converted into other polyamines, such as spermidine and spermine. In cancer, arginase and ODC activity are often upregulated, diverting arginine toward polyamine production to sustain rapid proliferation [254]. During colorectal tumorigenesis, the urea cycle becomes progressively activated. This activation can be driven by the absence of gut microbes with ureolytic capacity (e.g. *Bifidobacterium*), leading to the accumulation of intestinal urea. Elevated urea not only promotes colorectal cancer initiation but also skews macrophages toward a pro-tumoral phenotype through increased polyamine production [255]. At the same time, microbial production of polyamines in the gut provides an additional source that contributes to elevated systemic levels. Together, these pathways create a metabolic network that strongly supports tumour growth and survival. In the CRC mouse model, reduced arginine catabolism in the intestinal microbiota leads to elevated arginine levels in the TME. This promotes nitric oxide (NO) production and polyamine synthesis in tumour tissues, driving angiogenesis, M2 macrophage polarisation, and activation of the Wnt/β-catenin pathway, ultimately accelerating CRC progression [256]. Fan et al. found that germ-free mice have elevated colonic polyamine levels. Mechanistically, *Lactobacillus murinus* secretes small RNAs in extracellular vesicles that suppress host polyamine metabolism by targeting key enzymes. Notably, CRC in mice and humans is associated with reduced bacterial small RNAs and increased polyamines. These findings reveal a microbiota-mediated mechanism controlling host polyamine metabolism [257].

Despite their growth-promoting functions, polyamines act as a double-edged sword. When their catabolism is activated, polyamines can become toxic to cancer cells. Catabolic enzymes such as spermidine/spermine N1-acetyltransferase (SSAT) and polyamine oxidases (PAOX/SMOX) convert polyamines into hydrogen peroxide (H₂O₂) and reactive aldehydes. These byproducts generate oxidative stress, cause DNA damage, and disrupt mitochondrial function, ultimately leading to apoptosis [258]. Cancer cells are often addicted to making polyamines, which they need to grow. Hyperactivated SSAT makes them waste polyamines, increasing their need for recycling through the enzyme methylthioadenosine phosphorylase (MTAP). Affronti et al. showed that MTAP inhibition combined with SSAT upregulation synergistically increases apoptosis in prostate carcinoma models and patient explants [259]. Moreover, Bi et al. identified polyamines as a ferroptosis promoter functioning in an H₂O₂-dependent manner. Iron overload activates Wnt/Myc signalling, upregulating ODC1 and boosting polyamine synthesis, creating a positive feedback loop that amplifies ferroptosis. Ferroptotic cells also release polyamine-rich extracellular vesicles, sensitising adjacent cells and spreading ferroptosis in tumours. Moreover, polyamine supplementation increases cancer cell sensitivity to radio- and chemotherapy by inducing ferroptosis. Since cancer cells often have elevated polyamine pools, targeting this pathway offers a promising therapeutic strategy [260].

During polyamine synthesis, formate can be generated as a byproduct. This pathway begins with the deamination of methionine to form SAM, which then donates a propylamine group to polyamine synthesis, creating 5-methylthioadenosine (MTA), which can be released or recycled into adenine, methionine, and formate. Formate is the simplest carboxylic acid anion, derived from formic acid, supporting purine and thymidine synthesis [261]. Elevated systemic formate (from microbiota or tumour metabolism itself) can therefore feed tumour growth. High formate levels have been linked to chemotherapy resistance in lung cancer and to certain bacteria, including *Leuconostoc lactis* and *Eubacterium siraeum*. In CRC, increased *Fusobacterium nucleatum* (including clinical isolates) similarly drives chemoresistance through formate. High production of formic acid, lactic acid, and acetic acid was also observed in a clinical isolate of *Bacteroides faecalis *[262]. Moreover, *F. nucleatum* produces hydrogen sulphide from sulphur-containing amino acids, promoting cancers like colorectal, liver, and lung cancer. In CRC cells, hydrogen sulphide increased inflammation, survival, and migration, while transcriptome analysis showed activation of autophagy and inflammatory pathways. In mice, *F. nucleatum* and L-cysteine accelerated tumour progression, upregulated autophagy genes, reduced probiotics, and enriched pathogenic microbes [263]. Some other gut bacteria produce hydrogen sulphide through cysteine desulfurase–catalysed degradation of cysteine. The first cysteine desulfurases were identified in *Escherichia coli*, and since then, similar reactions have been described in common colonic bacteria, including *Clostridium*, *Enterobacter*, *Klebsiella*, *Streptococcus*, and sulphate-reducing *Desulfovibrio*. Exogenous hydrogen sulphide was shown to inhibit apoptosis in colon cancer cells. Consistently, studies on gut microbiota in patients with colonic adenomas and CRC reveal increased abundance of sulphur-producing bacteria such as *Bilophila*, *Desulfovibrio*, *Bilophila wadsworthia*, and *F. nucleatum *[264].

Urolithin A (uroA) is a microbial metabolite produced from ellagic acid and ellagitannins by certain gut bacteria (e.g., *Eggerthellaceae* family, *Enterocloster* spp.). Inducible uroC dehydroxylase (ucd) operon is a key for uroA production. Metagenomic and *ex vivo* analyses confirm that only microbiota actively transcribing ucd generate uroA, explaining inter-individual differences in urolithin metabolism [265]. UroA has gained attention because it can directly influence cancer cell fate. For example, uroA triggers cell cycle arrest and apoptosis in colorectal cancer cells by suppressing BCL-2, upregulating expression of the pro-apoptotic proteins p53 and p21, and enhancing ROS production [266]. Moreover, uroA inhibited the interaction between p53 and MDM2, inhibiting MDM2-mediated p53 polyubiquitination and degradation [267]. Finally, by dampening pro-inflammatory signalling pathways, uroA reduces the tumour-supportive environment. Mechanistically, uroA promotes TFEB nuclear localisation, enhances mitophagy, and limits IL-6 and TNF-α production and inflammation from damaged mitochondria, ultimately inhibiting tumour growth in breast cancer models [268].

## Microbes and cancer treatment

Modulation of cell death pathways is a key mechanism by which tumour cells develop drug resistance. In CRC, *F. nucleatum* activates autophagy by targeting TLR4, MYD88, and specific microRNAs, creating a molecular network that contributes to chemotherapy resistance [52, 269]. Analogously, *Peptostreptococcus stomatis*, enriched in CRC patients, activates the ERBB2-MAPK pathway, promoting tumour growth and reducing the efficacy of EGFR and BRAF inhibitors, thereby facilitating resistance to targeted therapies [270]. Other mechanisms include intratumoral *Gammaproteobacteria* (e.g., *E. coli K12*, *Klebsiella pneumoniae*, *Klebsiella oxytoca*, *Mycoplasma hyorhinis, Salmonella enterica*) metabolising gemcitabine into its inactive form. Accordingly, in a colon cancer mouse model, intratumoral *Gammaproteobacteria* induced resistance to gemcitabine [271]. Colbert et al. demonstrated that L-lactate produced by tumour-resident *Lactobacillus iners* reprograms the metabolism of cervical cancer cells, enhancing the Warburg effect and glycolysis, as well as glutamate and galactose metabolism, thereby driving resistance to chemoradiation. Similarly, lactic acid bacteria correlate with outcomes in colorectal, lung, head and neck, and skin cancers. These findings suggest that tumour-associated lactic acid bacteria drive lactate signalling–mediated resistance and represent potential therapeutic targets across cancers [272]. Moreover, Battaglia et al. observed tumour immune evasion mediated by cancer-associated fibroblasts (CAF) infiltration and immune exclusion in tumours with higher microbial diversity [273].

Based on these facts, it would seem rational to use antibiotics. Antibiotics can exert anticancer effects through both direct and indirect mechanisms. Directly, antibiotics, such as minocycline, tigecycline, and doxycycline, can impair mitochondrial protein synthesis due to the bacterial origin of mitochondria. This disruption of mitochondrial function reduces ATP production, induces oxidative stress, and triggers apoptosis in rapidly dividing cancer cells [274]. Indirectly, antibiotics can target tumour-associated bacteria that promote chemoresistance or tumour survival. For example, metronidazole reduced intratumoral *F. nucleatum* and suppressed cancer cell growth in CRC mouse models. Notably, human colorectal cancers colonised by *F. nucleatum* and its associated microbes, including *Bacteroides*, *Selenomonas*, and *Prevotella*, retain this microbial community in distal metastases, indicating stability of the tumour-promoting microbiome across primary and metastatic sites [275]. Notably, a key translational finding revealed that coadministration of the antibiotic ciprofloxacin effectively reversed gemcitabine resistance in mice [271].

On the other hand, several bacteria, including genetically modified strains of *Clostridium*, *Salmonella*, and *Bacillus Calmette-Guerin* (BCG), an attenuated strain of *Mycobacterium bovis*, are being investigated or used to trigger the elimination of cancer cells by directly causing cell death or by stimulating the immune system. BCG triggers caspase-independent cell death in carcinoma cells and promotes the release of an endogenous damage-associated molecular patterns (DAMPs) biomolecule HMGB1 [276]. HMGB1 triggers neutrophil extracellular trap (NETs) formation through interactions with Toll-like receptor 4 (TLR4) [277]. Hypoxic areas in solid tumours provide a niche for tumour-specific colonisation by anaerobic *Clostridium* species. *Clostridium novyi*-NT and *C. sporogenes* have shown promise in preclinical and early clinical models (NCT03435952, NCT0192468). Although their single-agent anti-tumour effects are modest, they can be used as a tumour-selective gene delivery system. Genetic engineering (via CRISPR–Cas9 or allele-coupled exchange) allows *Clostridium* to deliver therapeutic genes, such as prodrug-activating enzymes or express immunomodulators (e.g., IL-2, TNF-α), enhancing immunotherapy while reducing systemic toxicity [278]. *Salmonella enterica* can kill cancer cells through direct mechanisms, such as activating apoptosis and pyroptosis, and indirectly by stimulating the immune system and inhibiting tumour blood supply. Tumours contain both oxygen-poor and oxygen-rich regions, limiting the use of obligate anaerobes like *Clostridium*, which survive only in anoxic zones. In contrast, facultative anaerobes such as *Salmonella enterica* can colonise all tumour regions, including normoxic areas, making them more effective for targeting small and metastatic tumours. Its ability to selectively colonise tumours and deliver therapeutic agents also contributes to its effectiveness, making it a promising tool in bacterial-mediated cancer therapy [279, 280]. Moreover, *Salmonella enterica* serovar *Typhimurium* (*S. typhi*) selectively grows in tumours and reduces P-glycoprotein (P-gp) expression in cancer cells via its effector SipA through a pathway involving caspase-3. Mimicking this, gold nanoparticles coated with SipA were designed. These particles accumulate in tumours, lower P-gp expression, and enhance chemotherapy efficacy, overcoming multidrug resistance [281]. It was also demonstrated that *Salmonella* can deliver the cytosine deaminase gene to tumour tissue, where it remains functional, converting 5-fluorocytosine to the active drug 5-fluorouracil [282]. *Salmonella* can serve as a delivery vehicle for anticancer drugs or pro-apoptotic genes to target tumours [283]. Attenuated *S. typhi* shows potential as a cancer therapy by depleting asparagine in the TME via its asparaginase (ansB) activity, which suppresses tumour growth. However, this asparagine depletion also induces T-cell metabolic arrest by destabilizing c-Myc, limiting T-cell responses and the potential for immune memory or checkpoint blockade therapies. Using an *S. typhi* mutant with ansB deletion (∆ansB) restored T-cell function while still effectively reducing tumour growth [284]. The only FDA-approved bacterial therapy for cancer is BCG, which is used to treat non–muscle invasive bladder cancer (NMIBC). On April 22, 2024, the FDA approved nogapendekin alfa inbakicept-pmln (Anktiva, Altor BioScience, LLC) in combination with BCG for adults with BCG-unresponsive NMIBC with carcinoma *in situ*, with or without papillary tumours. The efficacy of Anktiva was evaluated in the QUILT-3.032 trial (NCT0302285).

Interestingly, some bacteria can directly take up extracellular DNA from their surroundings. This process represents one form of horizontal gene transfer (HGT); the exchange of genetic material between organisms outside of traditional parent-to-offspring inheritance. HGT is widespread among microbes but can also occur between microbes and higher organisms, including both microbe-to-eukaryote and eukaryote-to-microbe transfer. Cooper et al. advanced this concept by creating *Acinetobacter baylyi* strains capable of identifying oncogenic mutations in human DNA. This non-pathogenic bacterium can acquire DNA through HGT. The researchers modified it to gain drug resistance only when it acquired DNA containing a cancer-associated oncogene mutation, not the wild-type sequence. The engineered bacteria successfully detected their target in cell cultures and in mice with CRC, demonstrating promising diagnostic and tumour-targeting potential [285].

## Conclusions and future direction: preserving the good, eliminating the bad

Several bacterial species that are highly abundant in tumours, such as *Escherichia coli*, *Klebsiella pneumoniae*, *Staphylococcus aureus*, *Staphylococcus epidermidis*, *Pseudomonas* species, *Cutibacterium acnes* [286], and even traditionally beneficial taxa such as *Lactobacillus*, illustrate the dual nature of host–microbe interactions. In their natural niches, these microbes often provide health-promoting functions. For example, *E. coli* contributes to vitamin K and vitamin B12 production [287] and iron metabolism in the gut, as recent research shows that enterobactin (a siderophore) produced by *E. coli* can benefit host iron levels in the intestine by carrying iron into host cells [288]. *E. coli* also maintains a friendly environment for its anaerobic neighbours by consuming oxygen that enters the gut [287]. *S. epidermidis* and *C. acnes* support skin barrier function, and *Lactobacillus* species help maintain gut homeostasis, enhance epithelial defence, and produce metabolites such as lactic acid that prevent pathogen overgrowth. However, when these same microbes colonise tumour tissues, their effects can shift dramatically toward tumour promotion. For example, the enterobactin-binding protein Lipocalin 2 and its receptor SCL22A17, overproduced by cancer cells, can redirect iron flow from *E.coli* to cancer cells, supporting their survival and growth in iron-scarce environments [289, 290]. Strikingly, *Lactobacillus*, generally regarded as a probiotic, can promote tumour cell survival by producing high levels of lactate that fuel glycolysis, rewire metabolism, suppress cell death of cancer cells, and confer resistance to chemoradiation [272]. Similarly, AhR is a ligand-activated transcription factor environmental sensor that, when activated, influences T-regulatory cells (Tregs) by promoting their homing and enhancing their immune-suppressive functions, a process linked to maintaining gut homeostasis [291]. In cancer, immune evasion and therapy resistance are supported by AhR signalling. AhR activity is significantly modulated by microbial products, including Trp metabolites, such as kynurenic acid [146]. In epidermal keratinocytes, AhR represses nucleotide excision repair and apoptosis and contributes to UV-induced skin carcinogenesis [292]. Both pharmacological inhibition and genetic deletion of AhR significantly enhance erastin-induced ferroptosis by downregulating SLC7A11 and increasing lipid peroxidation. Moreover, AhR ligand indole-3-pyruvate (I3P) protected cells from ferroptosis in an AhR-dependent manner. These findings suggest that AhR transcriptionally regulates SLC family genes to control ferroptosis, highlighting its key role in preventing lipid oxidation and maintaining cell survival under oxidative stress [293], which is undoubtedly desirable in the intestine but problematic in cancer treatment. Nevertheless, the impact of AhR activation in cancer is highly ligand-, cell type-, and context-dependent.

Furthermore, P-gp protects the intestinal epithelium by exporting xenobiotics and mediating immune–barrier communication. Recent findings show that specific gut microbes—mainly *Clostridia* and *Bacilli*—induce P-gp expression through synergistic actions of SCFAs and secondary BAs. In ulcerative colitis, reduced P-gp levels correlate with loss of these metabolites and impaired epithelial anti-inflammatory signalling. In cancer, high P-gp expression contributes to chemotherapy resistance. Physiological levels of butyrate significantly increased P-gp expression and activity *in vitro*, while acetate and propionate had no effect. Notably, butyrate alone was sufficient to induce P-gp expression in germ-free mice [294]. Therefore, therapeutic use of butyrate-producing bacteria in the TME should be combined with local administration of P-gp inhibitors to prevent enhanced drug efflux and maintain treatment efficacy. Together, these examples highlight that bacteria beneficial in the gut or skin may adopt tumour-supportive roles when present in the TME.

Tumour-resident microbes can drive cancer progression and drug resistance, yet systemic antibiotics are problematic because they disrupt the gut microbiome that supports immunity and metabolic health. Emerging strategies may focus on selective elimination of harmful bacteria in the TME while preserving gut commensals and their metabolites, supporting survival of intestinal cells during anticancer treatment, and preventing mucositis or acute radiation syndrome (e.g., indole-3-propionic acid (IPA) protects against radiation-induced toxicity by preserving acyl-CoA–binding protein function [295]). Wang et al. developed a liposome-encapsulated silver–tinidazole complex that selectively targets *F. nucleatum*. This approach triggered the release of tumour-specific microbial neoantigens and enhanced CD8⁺ T-cell infiltration, effectively transforming immune-cold tumours into immune-hot ones and promoting immune recognition and killing of cancer cells [296]. Other possible approaches include localised antibiotic delivery via nanoparticles and hydrogels [297, 298], bacteriophage therapy [299] tailored to tumour-associated strains (e.g., *Fusobacterium nucleatum*, *Lactobacillus iners*), inhibitors of bacterial enzymes such as cytidine deaminase that inactivate chemotherapeutics, or engineered bacteria supporting killing of cancer cells by the immune system. Immunotherapy is highly effective in blood cancers but less successful in solid tumours. To address this, Vincent et al. engineered probiotic *E. coli* that selectively colonise tumours and release synthetic antigens, effectively “tagging” them for chimeric antigen receptor (CAR) T-cells programmed to recognise these tags. This probiotic-guided CAR-T system safely directed immune attacks at solid tumours and enhanced therapeutic efficacy in breast and colon cancer models [300]. Additional options involve engineered butyrate- or IAA-producing bacteria improving butyrate and IAA bioavailability, providing localised anti-cancer effects [301] or improving the efficacy of anticancer therapy [302, 303]. Accordingly, *Roseburia intestinalis* sensitised colorectal cancer to radiotherapy through the butyrate/OR51E1/RALB axis, facilitating autophagy-related cell death [304].

**Fig. 3:**
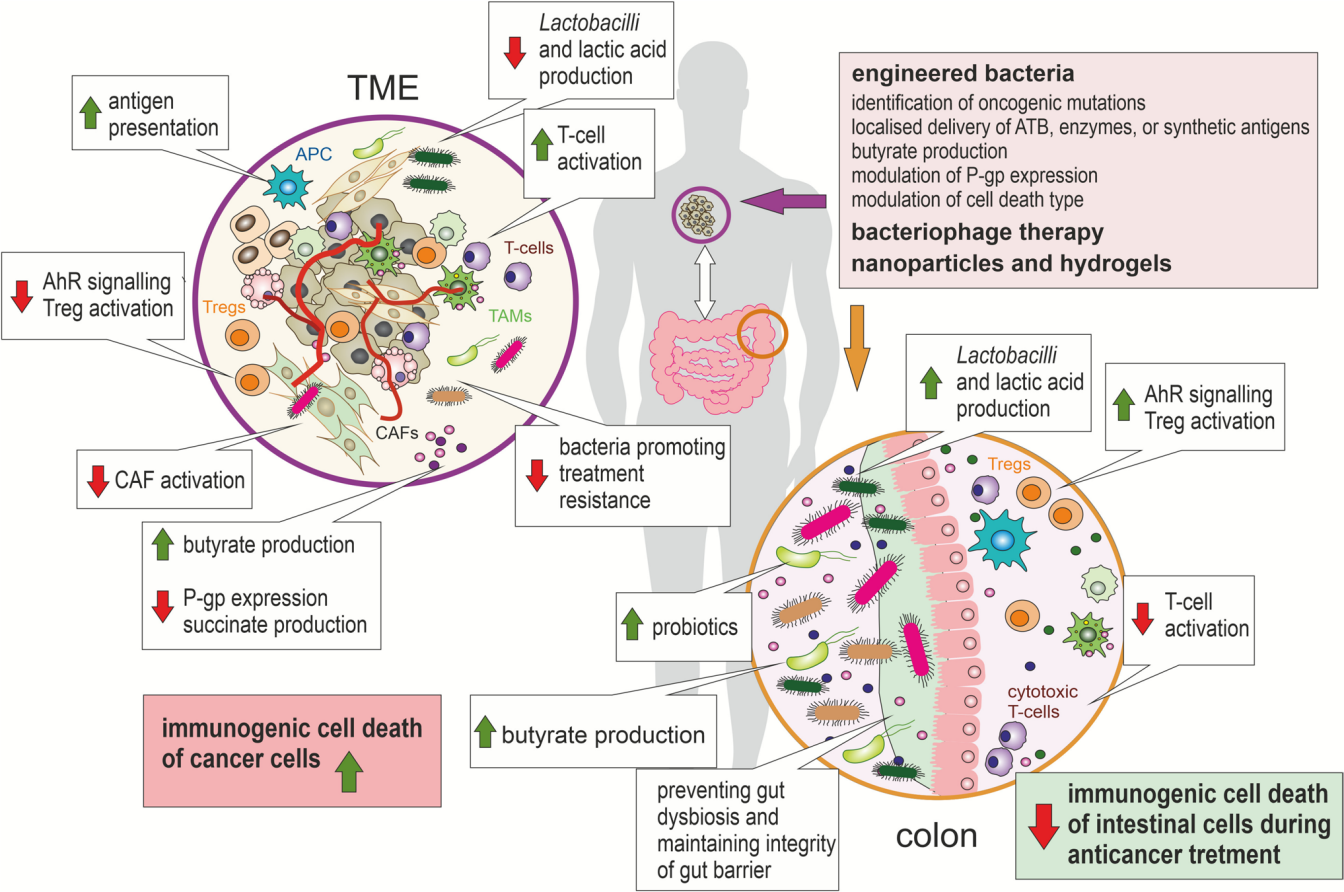
Microbiome-based approaches to enhance immunogenic cell death of cancer cells and protect the gut barrier during cancer therapy. The main goal of anticancer therapy should be to trigger immunogenic cell death (ICD) in tumour cells to promote systemic antitumour immunity and the abscopal effect, while simultaneously minimising ICD in intestinal epithelial cells to prevent excessive inflammation, autoimmune attacks, and maintain the integrity of the gut barrier. The term ICD is used for all types of cell death that can initiate an adaptive immune response against dead-cell–associated antigens. A promising emerging strategy is to harness microbes and their metabolites to identify and modulate tumour sensitivity to specific cell death pathways. Systematic analysis of microbial influences within the tumour microenvironment (TME) could enable selective activation or enhancement of defined cell death mechanisms, improving the precision and efficacy of cancer therapy. CAF = Cancer Associated Fibroblasts; APC = Antigen-Presenting Cells

A new promising therapeutic concept involves harnessing microbes and their metabolites to identify and modulate tumour sensitivity to specific forms of cell death. By systematically analysing microbial influences within the TME, it may become possible to selectively induce or amplify particular cell death pathways, thereby improving therapeutic precision. For instance, as mentioned in the previous text, *F. nucleatum* and *S. typhi* have been shown to enhance autophagy-related signalling, suggesting that their presence could be exploited to shift the balance and trigger autophagy-dependent cell death when combined with autophagy inducers under controlled conditions. Similarly, *E. coli*, *Pseudomonas aeruginosa*, polyamine metabolites, butyrate, or IAA can indicate tumour susceptibility to ferroptosis. On the other hand, the presence of bacteria expressing nicotinamidase (PncA) may confer resistance to NAMPT inhibitors and parthanatos. Moreover, microbes and metabolites such as *E. coli, Chlamydia trachomatis*, deoxycholic acid (DCA), succinate, and lactate are often associated with resistance to apoptosis or ferroptosis [305], and their elimination may support the anticancer effect of therapy. The main goal of anticancer therapy should be to trigger immunogenic cell death (ICD) in tumour cells to promote systemic antitumour immunity and the abscopal effect, while simultaneously minimising ICD in intestinal epithelial cells to prevent excessive inflammation and maintain the integrity of the gut barrier (Fig. 3). Unlike non-immunogenic death, ICD alerts and activates the immune system through the release of DAMPs, such as ATP, calreticulin, and HMGB1. These signals promote dendritic cell maturation, antigen presentation, and activation of cytotoxic CD8⁺ T-cells, thereby linking cell death to anti-tumour immunity. To optimise this approach, artificial intelligence (AI) and machine learning could be employed to integrate multi-omics data, including microbiome composition, metabolomic signatures, and tumour transcriptomics, to predict which microbial profiles sensitise or protect tumours from specific cell death pathways. Such models could guide the design of personalised microbe-guided cell death therapies. Ultimately, combining AI-driven prediction with microbial engineering holds the potential to reprogram tumour vulnerability with metabolic precision and spatial selectivity, offering a transformative strategy to overcome therapeutic resistance in cancer treatment.

## References

[1] Hanahan, D. (2022). Hallmarks of Cancer: New Dimensions. *Cancer Discovery,**12*, 31–46. 35022204 10.1158/2159-8290.CD-21-1059

[2] Labi, V., & Erlacher, M. (2015). How cell death shapes cancer. *Cell Death & Disease,**6*, Article e1675–e1675. 25741600 10.1038/cddis.2015.20 PMC4385913

[3] Sun, Z., et al. (2024). Immune-related cell death index and its application for hepatocellular carcinoma. *Npj Precision Oncology,**8*, Article 194. 39245753 10.1038/s41698-024-00693-9 PMC11381516

[4] Li, Y., et al. (2021). Review: Effect of gut microbiota and its metabolite SCFAs on radiation-induced intestinal injury. *Frontiers in Cellular and Infection Microbiology,**11*, Article 577236. 34307184 10.3389/fcimb.2021.577236 PMC8300561

[5] Li, Y., et al. (2020). Alterations of the gut microbiome composition and lipid metabolic profile in radiation enteritis. *Frontiers in Cellular and Infection Microbiology,**10*, Article 541178. 33194790 10.3389/fcimb.2020.541178 PMC7609817

[6] Patankar, J. V., & Becker, C. (2020). Cell death in the gut epithelium and implications for chronic inflammation. *Nature Reviews Gastroenterology & Hepatology,**17*, 543–556. 32651553 10.1038/s41575-020-0326-4

[7] Delgado, M. E., Grabinger, T., & Brunner, T. (2016). Cell death at the intestinal epithelial front line. *FEBS Journal,**283*, 2701–2719. 26499289 10.1111/febs.13575

[8] Chen, J., et al. (2025). Low-dose irradiation of the gut improves the efficacy of PD-L1 blockade in metastatic cancer patients. *Cancer Cell,**43*, 361-379.e10. 40068595 10.1016/j.ccell.2025.02.010 PMC11907695

[9] Anderson, C. J., et al. (2021). Microbes exploit death-induced nutrient release by gut epithelial cells. *Nature,**596*, 262–267. 34349263 10.1038/s41586-021-03785-9

[10] Upadhyayula, P. S., et al. (2023). Dietary restriction of cysteine and methionine sensitizes gliomas to ferroptosis and induces alterations in energetic metabolism. *Nature Communications,**14*, 1187. 36864031 10.1038/s41467-023-36630-w PMC9981683

[11] Vega, A. A., et al. (2023). Methionine-producing tumor micro(be) environment fuels growth of solid tumors. *Cellular Oncology,**46*, 1659–1673. 37318751 10.1007/s13402-023-00832-7 PMC10697899

[12] Green, D. R. (2024). Cell death: Revisiting the roads to ruin. *Developmental Cell,**59*, 2523–2531. 39378838 10.1016/j.devcel.2024.08.008 PMC11469552

[13] Galluzzi, L., Kepp, O., Zitvogel, L., Tang, D., & Kroemer, G. (2025). Cancer cell death: Cell-autonomous and immunogenic dimensions. *Cancer Cell,**S1535–6108*(25), 00539–00542. 10.1016/j.ccell.2025.12.005 41478276

[14] Eskander, G., Abdelhamid, S. G., Wahdan, S. A., & Radwan, S. M. (2025). Insights on the crosstalk among different cell death mechanisms. *Cell Death Discovery,**11*, 1–22. 39929794 10.1038/s41420-025-02328-9 PMC11811070

[15] Galluzzi, L., et al. (2018). Molecular mechanisms of cell death: Recommendations of the Nomenclature Committee on Cell Death 2018. *Cell Death and Differentiation,**25*, 486–541. 29362479 10.1038/s41418-017-0012-4 PMC5864239

[16] Barnhart, B. C., Alappat, E. C., & Peter, M. E. (2003). The CD95 type I/type II model. *Seminars in Immunology,**15*, 185–193. 14563117 10.1016/s1044-5323(03)00031-9

[17] Jost, P. J., et al. (2009). XIAP discriminates between type I and type II FAS-induced apoptosis. *Nature,**460*, 1035–1039. 19626005 10.1038/nature08229 PMC2956120

[18] Yin, X. M., et al. (1999). Bid-deficient mice are resistant to Fas-induced hepatocellular apoptosis. *Nature,**400*, 886–891.10476969 10.1038/23730

[19] Hao, Z., & Mak, T. W. (2010). Type I and type II pathways of Fas-mediated apoptosis are differentially controlled by XIAP. *Journal of Molecular Cell Biology,**2*, 63–64. 19837685 10.1093/jmcb/mjp034

[20] Vanden Berghe, T., Linkermann, A., Jouan-Lanhouet, S., Walczak, H., & Vandenabeele, P. (2014). Regulated necrosis: The expanding network of non-apoptotic cell death pathways. *Nature Reviews Molecular Cell Biology,**15*, 135–147. 24452471 10.1038/nrm3737

[21] Li, Y., et al. (2021). Inflammasomes as therapeutic targets in human diseases. *Sig Transduct Target Ther,**6*, 247. 10.1038/s41392-021-00650-z PMC8249422 34210954

[22] Schachter, J., et al. (2025). Gasdermin D mediates a fast transient release of ATP after NLRP3 inflammasome activation before ninjurin 1-induced lytic cell death. *Cell Reports*. 10.1016/j.celrep.2025.115233 40010300 10.1016/j.celrep.2025.115233

[23] Nagata, S., & Tanaka, M. (2017). Programmed cell death and the immune system. *Nature Reviews. Immunology,**17*, 333–340. 28163302 10.1038/nri.2016.153

[24] Rogers, C., et al. (2019). Gasdermin pores permeabilize mitochondria to augment caspase-3 activation during apoptosis and inflammasome activation. *Nature Communications,**10*, Article 1689. 30976076 10.1038/s41467-019-09397-2 PMC6459836

[25] Gurung, P., Lukens, J. R., & Kanneganti, T.-D. (2015). Mitochondria: Diversity in the regulation of the NLRP3 inflammasome. *Trends in Molecular Medicine,**21*, 193–201. 25500014 10.1016/j.molmed.2014.11.008 PMC4352396

[26] Huang, J., Hong, W., Wan, M., & Zheng, L. (2022). Molecular mechanisms and therapeutic target of NETosis in diseases. *MedComm (2020),**3*, Article e162. 36000086 10.1002/mco2.162 PMC9390875

[27] Wise, A. D., et al. (2025). Mitochondria sense bacterial lactate and drive release of neutrophil extracellular traps. *Cell Host & Microbe,**33*, 341-357.e9. 40020664 10.1016/j.chom.2025.02.003 PMC11955204

[28] Ronchetti, L., et al. (2021). Neutrophil extracellular traps in cancer: Not only catching microbes. *Journal of Experimental & Clinical Cancer Research,**40*, Article 231. 34261496 10.1186/s13046-021-02036-z PMC8281578

[29] Berends, E. T. M., et al. (2010). Nuclease expression by *Staphylococcus aureus* facilitates escape from neutrophil extracellular traps. *Journal of Innate Immunity,**2*, 576–586. 20829609 10.1159/000319909 PMC2982853

[30] Liu, Y., Stockwell, B. R., Jiang, X., & Gu, W. (2025). P53-regulated non-apoptotic cell death pathways and their relevance in cancer and other diseases. *Nature Reviews. Molecular Cell Biology,**26*, 600–614. 40204927 10.1038/s41580-025-00842-3 PMC13014097

[31] Zhang, C., Liu, X., Jin, S., Chen, Y., & Guo, R. (2022). Ferroptosis in cancer therapy: A novel approach to reversing drug resistance. *Molecular Cancer,**21*, Article 47. 35151318 10.1186/s12943-022-01530-y PMC8840702

[32] Xie, W., Agarwal, S., & Yu, J. (2023). Ferroptosis: The vulnerability within a cancer monster. *Journal of Clinical Investigation*, *133*, Article e170027. 10.1172/JCI170027 10.1172/JCI170027 PMC10178832 37183818

[33] Koeberle, S. C., Kipp, A. P., Stuppner, H., & Koeberle, A. (2023). Ferroptosis-modulating small molecules for targeting drug-resistant cancer: Challenges and opportunities in manipulating redox signaling. *Medicinal Research Reviews,**43*, 614–682. 36658724 10.1002/med.21933 PMC10947485

[34] Yeung, Y. W. S., Ma, Y., Deng, Y., Khoo, B. L., & Chua, S. L. (2024). Bacterial iron siderophore drives tumor survival and ferroptosis resistance in a biofilm-tumor spheroid coculture model. *Advanced Science (Weinheim, Germany),**11*, Article e2404467. 10.1002/advs.202404467 PMC11496991 39135304

[35] Ellermann, M., & Arthur, J. C. (2017). Siderophore-mediated iron acquisition and modulation of host-bacterial interactions. *Free Radical Biology & Medicine,**105*, 68–78. 27780750 10.1016/j.freeradbiomed.2016.10.489 PMC5401654

[36] Zhao, Q., et al. (2025). Lactiplantibacillus plantarum -derived extracellular vesicles alleviate acute lung injury by inhibiting ferroptosis of macrophages. *Journal of Nanobiotechnology,**23*, 307. 40269965 10.1186/s12951-025-03405-y PMC12016285

[37] Furumoto, H., et al. (2016). 10-oxo-trans-11-octadecenoic acid generated from linoleic acid by a gut lactic acid bacterium *Lactobacillus plantarum* is cytoprotective against oxidative stress. *Toxicology and Applied Pharmacology,**296*, 1–9. 26879219 10.1016/j.taap.2016.02.012

[38] Jin, Z., et al. (2023). The gut metabolite 3-hydroxyphenylacetic acid rejuvenates spermatogenic dysfunction in aged mice through GPX4-mediated ferroptosis. *Microbiome,**11*, 212. 37752615 10.1186/s40168-023-01659-y PMC10523725

[39] Deng, F., et al. (2021). The gut microbiota metabolite capsiate promotes Gpx4 expression by activating TRPV1 to inhibit intestinal ischemia reperfusion-induced ferroptosis. *Gut Microbes,**13*, 1–21. 33779497 10.1080/19490976.2021.1902719 PMC8009132

[40] Wen, W., et al. (2023). PUFAs add fuel to Crohn’s disease-associated AIEC-induced enteritis by exacerbating intestinal epithelial lipid peroxidation. *Gut Microbes,**15*, Article 2265578. 37800577 10.1080/19490976.2023.2265578 PMC10561586

[41] Shao, D.-H., Yao, Y.-F., & Wang, D.-N. (2025). Ferroptosis and iron-based therapies in *Pseudomonas aeruginosa* infections: From pathogenesis to treatment. *Virulence,**16*, Article 2553787. 40911411 10.1080/21505594.2025.2553787 PMC12416177

[42] Gupta, G., et al. (2025). Parthanatos and apoptosis: Unraveling their roles in cancer cell death and therapy resistance. *EXCLI Journal,**24*, 351–380. 40166425 10.17179/excli2025-8251 PMC11956527

[43] Murata-Kamiya, N., & Hatakeyama, M. (2022). *Helicobacter pylori*-induced DNA double-stranded break in the development of gastric cancer. *Cancer Science,**113*, 1909–1918. 35359025 10.1111/cas.15357 PMC9207368

[44] Cuevas-Ramos, G., et al. (2010). *Escherichia coli* induces DNA damage in vivo and triggers genomic instability in mammalian cells. *Proceedings of the National Academy of Sciences of the United States of America,**107*, 11537–11542. 20534522 10.1073/pnas.1001261107 PMC2895108

[45] Zhu, L., et al. (2017). Contribution of Secreted NADase and Streptolysin O to the Pathogenesis of Epidemic Serotype M1 *Streptococcus pyogenes* Infections. *The American Journal of Pathology,**187*, 605–613. 28034602 10.1016/j.ajpath.2016.11.003 PMC5397666

[46] Tsapieva, A. N., et al. (2025). Studying the Oncolytic Activity of Streptococcus pyogenes Strains Against Hepatoma, Glioma, and Pancreatic Cancer In Vitro and In Vivo. *Microorganisms,**13*, 76. 39858844 10.3390/microorganisms13010076 PMC11767589

[47] Xie, J., Yang, Y., Gao, Y., & He, J. (2023). Cuproptosis: Mechanisms and links with cancers. *Molecular Cancer,**22*, Article 46. 36882769 10.1186/s12943-023-01732-y PMC9990368

[48] Nakagawa, T., et al. (2005). Cyclophilin D-dependent mitochondrial permeability transition regulates some necrotic but not apoptotic cell death. *Nature,**434*, 652–658. 15800626 10.1038/nature03317

[49] Raudenska, M., Balvan, J., & Masarik, M. (2021). Crosstalk between autophagy inhibitors and endosome-related secretory pathways: A challenge for autophagy-based treatment of solid cancers. *Molecular Cancer,**20*, Article 140. 34706732 10.1186/s12943-021-01423-6 PMC8549397

[50] Cho, T. J., et al. (2014). Porphyromonas gingivalis-induced autophagy suppresses cell proliferation through G1 arrest in oral cancer cells. *Archives of Oral Biology,**59*, 370–378. 24606908 10.1016/j.archoralbio.2014.01.001

[51] Yu, T., et al. (2017). *Fusobacterium nucleatum* promotes chemoresistance to colorectal cancer by modulating autophagy. *Cell,**170*, 548-563.e16. 28753429 10.1016/j.cell.2017.07.008 PMC5767127

[52] Liu, Y., et al. (2021). *Fusobacterium nucleatum* confers chemoresistance by modulating autophagy in oesophageal squamous cell carcinoma. *British Journal of Cancer,**124*, 963–974. 33299132 10.1038/s41416-020-01198-5 PMC7921654

[53] Chen, Y., et al. (2020). *Fusobacterium nucleatum* promotes metastasis in colorectal cancer by activating autophagy signaling via the upregulation of CARD3 expression. *Theranostics,**10*, 323–339. 31903123 10.7150/thno.38870 PMC6929621

[54] Liu, B., et al. (2016). Blockage of autophagy pathway enhances *Salmonella* tumor-targeting. *Oncotarget,**7*, 22873–22882. 27013582 10.18632/oncotarget.8251 PMC5008408

[55] Lin, H.-J., et al. (2017). Cytolethal distending toxin enhances radiosensitivity in prostate cancer cells by regulating autophagy. *Frontiers in Cellular and Infection Microbiology,**7*, Article 223. 28642840 10.3389/fcimb.2017.00223 PMC5462984

[56] Wang, S., et al. (2023). The mitophagy pathway and its implications in human diseases. *Sig Transduct Target Ther,**8*, 304. 10.1038/s41392-023-01503-7 PMC10427715 37582956

[57] Liu, S., Yao, S., Yang, H., Liu, S., & Wang, Y. (2023). Autophagy: Regulator of cell death. *Cell Death & Disease,**14*, Article 648. 37794028 10.1038/s41419-023-06154-8 PMC10551038

[58] Guilbaud, E., & Galluzzi, L. (2023). Adaptation to MOMP drives cancer persistence. *Cell Research,**33*, 93–94. 36163426 10.1038/s41422-022-00729-4 PMC9892491

[59] Waguia Kontchou, C., et al. (2022). *Chlamydia trachomatis* inhibits apoptosis in infected cells by targeting the pro-apoptotic proteins Bax and Bak. *Cell Death and Differentiation,**29*, 2046–2059. 35397654 10.1038/s41418-022-00995-0 PMC9525694

[60] González, E., et al. (2014). *Chlamydia* infection depends on a functional MDM2-p53 axis. *Nature Communications,**5*, Article 5201. 25392082 10.1038/ncomms6201 PMC4243245

[61] Wang, X., et al. (2025). *Chlamydia trachomatis* regulates ferroptosis through the p53/SLC7A11 pathway to promote reproduction. *Microbes and Infection,**27*, Article 105505. 40252953 10.1016/j.micinf.2025.105505

[62] Vanden Berghe, T., Kaiser, W. J., Bertrand, M. J. M., & Vandenabeele, P. (2015). Molecular crosstalk between apoptosis, necroptosis, and survival signaling. *Molecular & Cellular Oncology,**2*, Article e975093. 27308513 10.4161/23723556.2014.975093 PMC4905361

[63] Osbron, C. A., Lawson, C., Hanna, N., Koehler, H. S., & Goodman, A. G. (2024). Caspase-8 activity mediates TNFα production and restricts *Coxiella burnetii* replication during murine macrophage infection. *Infection and Immunity,**92*, Article e00053-24. 38837340 10.1128/iai.00053-24 PMC11238558

[64] Melenotte, C., et al. (2016). B-cell non-Hodgkin lymphoma linked to *Coxiella burnetii*. *Blood,**127*, 113–121. 26463422 10.1182/blood-2015-04-639617

[65] Faugaret, D., et al. (2014). Granulomatous response to Coxiella burnetii, the agent of Q fever: The lessons from gene expression analysis. *Frontiers in Cellular and Infection Microbiology,**4*, 172. 25566510 10.3389/fcimb.2014.00172 PMC4266094

[66] Weinlich, R., Oberst, A., Beere, H. M., & Green, D. R. (2017). Necroptosis in development, inflammation and disease. *Nature Reviews. Molecular Cell Biology,**18*, 127–136. 27999438 10.1038/nrm.2016.149

[67] Ruiz, E. J., et al. (2019). LUBAC determines chemotherapy resistance in squamous cell lung cancer. *The Journal of Experimental Medicine,**216*, 450–465. 30642944 10.1084/jem.20180742 PMC6363428

[68] de Jong, M. F., Liu, Z., Chen, D., & Alto, N. M. (2016). *Shigella flexneri* suppresses NF-kB activation by inhibiting linear ubiquitin chain ligation. *Nature Microbiology,**1*, Article 16084. 27572974 10.1038/nmicrobiol.2016.84 PMC5010086

[69] Khodavirdipour, A., Jamshidi, F., Nejad, H. R., Zandi, M., & Zarean, R. (2021). To study the anti-cancer effects of *Shigella Flexneri* in AspC-1 pancreatic cancer cell line in approach to Bax and bcl-2 genes. *Journal of Gastrointestinal Cancer,**52*, 593–599. 32524303 10.1007/s12029-020-00433-9

[70] Zychlinsky, A., Prevost, M. C., & Sansonetti, P. J. (1992). *Shigella flexneri* induces apoptosis in infected macrophages. *Nature,**358*, 167–169. 1614548 10.1038/358167a0

[71] Willson, J. (2020). A matter of life and death for caspase 8. *Nature Reviews Molecular Cell Biology,**21*, 63. 31844186 10.1038/s41580-019-0201-8

[72] Fritsch, M., et al. (2019). Caspase-8 is the molecular switch for apoptosis, necroptosis and pyroptosis. *Nature,**575*, 683–687. 31748744 10.1038/s41586-019-1770-6

[73] Wang, Y., et al. (2018). GSDME mediates caspase-3-dependent pyroptosis in gastric cancer. *Biochemical and Biophysical Research Communications,**495*, 1418–1425. 29183726 10.1016/j.bbrc.2017.11.156

[74] Wang, Y., et al. (2017). Chemotherapy drugs induce pyroptosis through caspase-3 cleavage of a gasdermin. *Nature,**547*, 99–103. 28459430 10.1038/nature22393

[75] Wright, S. S., et al. (2024). A bacterial toxin co-opts caspase-3 to disable active gasdermin D and limit macrophage pyroptosis. *Cell Reports,**43*, Article 114004. 38522070 10.1016/j.celrep.2024.114004 PMC11095105

[76] Gao, J., et al. (2024). PANoptosis: Bridging apoptosis, pyroptosis, and necroptosis in cancer progression and treatment. *Cancer Gene Therapy,**31*, 970–983. 38553639 10.1038/s41417-024-00765-9 PMC11257964

[77] Malireddi, R. K. S., Kesavardhana, S., & Kanneganti, T.-D. (2019). ZBP1 and TAK1: Master regulators of NLRP3 inflammasome/pyroptosis, apoptosis, and necroptosis (PAN-optosis). *Frontiers in Cellular and Infection Microbiology,**9*, Article 406. 31850239 10.3389/fcimb.2019.00406 PMC6902032

[78] Tsai, M.-S., Chen, Y.-Y., Chen, W.-C., & Chen, M.-F. (2022). *Streptococcus mutans* promotes tumor progression in oral squamous cell carcinoma. *Journal of Cancer,**13*, 3358–3367. 36186905 10.7150/jca.73310 PMC9516012

[79] Yu, L., et al. (2022). The oral bacterium *Streptococcus mutans* promotes tumor metastasis by inducing vascular inflammation. *Cancer Science,**113*, 3980–3994. 35997541 10.1111/cas.15538 PMC9633306

[80] He, S., Chakraborty, R., & Ranganathan, S. (2022). Proliferation and apoptosis pathways and factors in oral squamous cell carcinoma. *International Journal of Molecular Sciences,**23*, Article 1562. 35163485 10.3390/ijms23031562 PMC8836072

[81] Han, J.-H., et al. (2024). NINJ1 mediates inflammatory cell death, PANoptosis, and lethality during infection conditions and heat stress. *Nature Communications,**15*, 1739. 38409108 10.1038/s41467-024-45466-x PMC10897308

[82] Wang, H., Guo, M., Wei, H., & Chen, Y. (2023). Targeting p53 pathways: Mechanisms, structures and advances in therapy. *Signal Transduction and Targeted Therapy,**8*, Article 92. 36859359 10.1038/s41392-023-01347-1 PMC9977964

[83] Liu, Y., & Gu, W. (2022). p53 in ferroptosis regulation: The new weapon for the old guardian. *Cell Death & Differentiation,**29*, 895–910. 35087226 10.1038/s41418-022-00943-y PMC9091200

[84] Jiang, L., et al. (2015). Ferroptosis as a p53-mediated activity during tumour suppression. *Nature,**520*, 57–62. 25799988 10.1038/nature14344 PMC4455927

[85] Rius-Pérez, S., Pérez, S., Toledano, M. B., & Sastre, J. (2022). p53 drives necroptosis via downregulation of sulfiredoxin and peroxiredoxin 3. *Redox Biology,**56*, Article 102423. 36029648 10.1016/j.redox.2022.102423 PMC9428851

[86] Wang, X., et al. (2024). The p53 target DRAM1 modulates calcium homeostasis and ER stress by promoting contact between lysosomes and the ER through STIM1. *Proceedings of the National Academy of Sciences,**121*, Article e2400531121. 10.1073/pnas.2400531121 PMC11441506 39292746

[87] Xiong, C., Ling, H., Hao, Q., & Zhou, X. (2023). Cuproptosis: P53-regulated metabolic cell death? *Cell Death & Differentiation,**30*, 876–884. 36755067 10.1038/s41418-023-01125-0 PMC10070433

[88] Cougnoux, A., et al. (2014). Bacterial genotoxin colibactin promotes colon tumour growth by inducing a senescence-associated secretory phenotype. *Gut,**63*, 1932–1942. 24658599 10.1136/gutjnl-2013-305257

[89] Dougherty, M. W., et al. (2023). The microbial genotoxin colibactin exacerbates mismatch repair mutations in colorectal tumors. *Neoplasia,**43*, Article 100918. 37499275 10.1016/j.neo.2023.100918 PMC10413156

[90] Iftekhar, A., et al. (2021). Genomic aberrations after short-term exposure to colibactin-producing E. coli transform primary colon epithelial cells. *Nature Communications*, *12*, Article 1003. 10.1038/s41467-021-21162-y 10.1038/s41467-021-21162-y PMC7881031 33579932

[91] Chagneau, C. V., et al. (2019). The polyamine spermidine modulates the production of the bacterial genotoxin colibactin. *M Sphere,**4*, Article e00414-19. 10.1128/mSphere.00414-19 PMC6796968 31578245

[92] Pugin, B., et al. (2017). A wide diversity of bacteria from the human gut produces and degrades biogenic amines. *Microbial Ecology in Health and Disease,**28*, 1353881. 28959180 10.1080/16512235.2017.1353881 PMC5614385

[93] Mirzarazi, M., et al. (2022). The OmpA of commensal Escherichia coli of CRC patients affects apoptosis of the HCT116 colon cancer cell line. *BMC Microbiology,**22*, 139. 35590263 10.1186/s12866-022-02540-y PMC9118694

[94] Gamallat, Y., et al. (2016). *Lactobacillus rhamnosus* induced epithelial cell apoptosis, ameliorates inflammation and prevents colon cancer development in an animal model. *Biomedicine & Pharmacotherapy,**83*, 536–541. 27447122 10.1016/j.biopha.2016.07.001

[95] Fareez, I. M., Lim, S. M., & Ramasamy, K. (2024). Chemoprevention by microencapsulated *Lactiplantibacillus Plantarum* LAB12 against orthotopic colorectal cancer mice is associated with apoptosis and anti-angiogenesis. *Probiotics and Antimicrobial Proteins,**16*, 99–112. 36508139 10.1007/s12602-022-10020-y

[96] Meng, X., Zhang, J., Wu, H., Yu, D., & Fang, X. (2020). *Akkermansia muciniphila* Aspartic Protease Amuc_1434* inhibits human colorectal cancer LS174T cell viability via TRAIL-mediated apoptosis pathway. *International Journal of Molecular Sciences,**21*, 3385. 32403433 10.3390/ijms21093385 PMC7246985

[97] Nishida, T., Naguro, I., & Ichijo, H. (2022). NAMPT-dependent NAD+ salvage is crucial for the decision between apoptotic and necrotic cell death under oxidative stress. *Cell Death Discovery,**8*, Article 195. 35410407 10.1038/s41420-022-01007-3 PMC9001718

[98] Ying, W., Alano, C. C., Garnier, P., & Swanson, R. A. (2005). NAD+ as a metabolic link between DNA damage and cell death. *Journal of Neuroscience Research,**79*, 216–223. 15562437 10.1002/jnr.20289

[99] Wu, Q.-J., et al. (2022). The sirtuin family in health and disease. *Sig Transduct Target Ther,**7*, 402. 10.1038/s41392-022-01257-8 PMC9797940 36581622

[100] Vaziri, H., et al. (2001). hSIR2(SIRT1) functions as an NAD-dependent p53 deacetylase. *Cell,**107*, 149–159. 11672523 10.1016/s0092-8674(01)00527-x

[101] Ong, A. L. C., & Ramasamy, T. S. (2018). Role of Sirtuin1-p53 regulatory axis in aging, cancer and cellular reprogramming. *Ageing Research Reviews,**43*, 64–80. 29476819 10.1016/j.arr.2018.02.004

[102] Motta, M. C., et al. (2004). Mammalian SIRT1 represses forkhead transcription factors. *Cell,**116*, 551–563. 14980222 10.1016/s0092-8674(04)00126-6

[103] Sarmah, D., et al. (2022). Sirtuin-1 - Mediated NF-κB pathway modulation to mitigate inflammasome signaling and cellular apoptosis is one of the neuroprotective effects of intra-arterial mesenchymal stem cell therapy following ischemic stroke. *Stem Cell Reviews and Reports,**18*, 821–838. 35112234 10.1007/s12015-021-10315-7

[104] Crowley, C. L., Payne, C. M., Bernstein, H., Bernstein, C., & Roe, D. (2000). The NAD+ precursors, nicotinic acid and nicotinamide protect cells against apoptosis induced by a multiple stress inducer, deoxycholate. *Cell Death and Differentiation,**7*, 314–326. 10745276 10.1038/sj.cdd.4400658

[105] Luo, G., et al. (2019). Sirt1 promotes autophagy and inhibits apoptosis to protect cardiomyocytes from hypoxic stress. *International Journal of Molecular Medicine,**43*, 2033–2043. 30864731 10.3892/ijmm.2019.4125 PMC6443335

[106] Lee, I. H., et al. (2008). A role for the NAD-dependent deacetylase Sirt1 in the regulation of autophagy. *Proceedings of the National Academy of Sciences,**105*, 3374–3379. 10.1073/pnas.0712145105 PMC2265142 18296641

[107] Wilk, A., et al. (2020). Extracellular NAD+ enhances PARP-dependent DNA repair capacity independently of CD73 activity. *Scientific Reports,**10*, 651. 31959836 10.1038/s41598-020-57506-9 PMC6971268

[108] Ying, W., Garnier, P., & Swanson, R. A. (2003). NAD+ repletion prevents PARP-1-induced glycolytic blockade and cell death in cultured mouse astrocytes. *Biochemical and Biophysical Research Communications,**308*, 809–813. 12927790 10.1016/s0006-291x(03)01483-9

[109] Iske, J., et al. (2024). NAD⁺ prevents septic shock‑induced death by non‑canonical inflammasome blockade and IL‑10 cytokine production in macrophages. *eLife*, *12*, Article RP88686. 10.7554/eLife.88686 10.7554/eLife.88686 PMC10942599 38372712

[110] Hong, Y., et al. (2014). NAD+ treatment prevents rotenone-induced apoptosis and necrosis of differentiated PC12 cells. *Neuroscience Letters,**560*, 46–50. 24304867 10.1016/j.neulet.2013.11.039

[111] Sundaram, B., et al. (2024). NLRC5 senses NAD+ depletion, forming a PANoptosome and driving PANoptosis and inflammation. *Cell,**187*, 4061-4077.e17. 38878777 10.1016/j.cell.2024.05.034 PMC11283362

[112] Hubert, S., et al. (2010). Extracellular NAD+ shapes the Foxp3+ regulatory T cell compartment through the ART2–P2X7 pathway. *Journal of Experimental Medicine,**207*, 2561–2568. 20975043 10.1084/jem.20091154 PMC2989765

[113] Rajman, L., Chwalek, K., & Sinclair, D. A. (2018). Therapeutic potential of NAD-boosting molecules: The in vivo evidence. *Cell Metabolism,**27*, 529–547. 29514064 10.1016/j.cmet.2018.02.011 PMC6342515

[114] Cambronne, X. A., & Kraus, W. L. (2020). Location, location, location: Compartmentalization of NAD+ synthesis and functions in mammalian cells. *Trends in Biochemical Sciences,**45*, 858–873. 32595066 10.1016/j.tibs.2020.05.010 PMC7502477

[115] Chellappa, K., et al. (2022). NAD precursors cycle between host tissues and the gut microbiome. *Cell Metabolism,**34*, 1947-1959.e5. 36476934 10.1016/j.cmet.2022.11.004 PMC9825113

[116] Hee, B., & Wells, J. M. (2021). Microbial regulation of host physiology by short-chain fatty acids. *Trends in Microbiology,**29*, 700–712. 33674141 10.1016/j.tim.2021.02.001

[117] Elangovan, S., et al. (2014). The niacin/butyrate receptor GPR109A suppresses mammary tumorigenesis by inhibiting cell survival. *Cancer Research,**74*, 1166–1178. 24371223 10.1158/0008-5472.CAN-13-1451 PMC3944627

[118] Han, X., & Simon, M. C. (2022). NAD+ regeneration drives cancer cell proliferation. *Nature Metabolism,**4*, 647–648. 35739398 10.1038/s42255-022-00586-w

[119] Li, Z., et al. (2022). Cancer cells depend on environmental lipids for proliferation when electron acceptors are limited. *Nature Metabolism,**4*, 711–723. 35739397 10.1038/s42255-022-00588-8 PMC10305743

[120] Luengo, A., et al. (2021). Increased demand for NAD+ relative to ATP drives aerobic glycolysis. *Molecular Cell,**81*, 691-707.e6. 33382985 10.1016/j.molcel.2020.12.012 PMC8315838

[121] Lauterwasser, J., et al. (2021). Hexokinases inhibit death receptor–dependent apoptosis on the mitochondria. *Proc Natl Acad Sci U S A,**118*, Article e2021175118. 34385311 10.1073/pnas.2021175118 PMC8379972

[122] Zheng, X., et al. (2021). ANGPTL4-mediated promotion of glycolysis facilitates the colonization of *Fusobacterium nucleatum* in colorectal cancer. *Cancer Research,**81*, 6157–6170. 34645607 10.1158/0008-5472.CAN-21-2273 PMC9397643

[123] Kaplan, C. W., et al. (2010). Fusobacterium nucleatum outer membrane proteins Fap2 and RadD induce cell death in human lymphocytes. *Infection and Immunity,**78*, 4773–4778. 20823215 10.1128/IAI.00567-10 PMC2976331

[124] Wang, B., et al. (2011). NAMPT overexpression in prostate cancer and its contribution to tumor cell survival and stress response. *Oncogene,**30*, 907–921. 20956937 10.1038/onc.2010.468

[125] Nelson, A. E., & Heske, C. M. (2025). Mechanisms of resistance to NAMPT inhibitors in cancer. *Cancer Drug Resistance*, *8*, Article 18. 10.20517/cdr.2024.216 10.20517/cdr.2024.216 PMC12059476 40342733

[126] Lucena-Cacace, A., Otero-Albiol, D., Jiménez-García, M. P., Peinado-Serrano, J., & Carnero, A. (2017). NAMPT overexpression induces cancer stemness and defines a novel tumor signature for glioma prognosis. *Oncotarget,**8*, 99514–99530. 29245920 10.18632/oncotarget.20577 PMC5725111

[127] Lv, H., et al. (2021). NAD+ metabolism maintains inducible PD-L1 expression to drive tumor immune evasion. *Cell Metabolism,**33*, 110-127.e5. 33171124 10.1016/j.cmet.2020.10.021

[128] Subedi, A., et al. (2021). Nicotinamide phosphoribosyltransferase inhibitors selectively induce apoptosis of AML stem cells by disrupting lipid homeostasis. *Cell Stem Cell,**28*, 1851-1867.e8. 34293334 10.1016/j.stem.2021.06.004

[129] Chowdhry, S., et al. (2019). NAD metabolic dependency in cancer is shaped by gene amplification and enhancer remodelling. *Nature,**569*, 570–575. 31019297 10.1038/s41586-019-1150-2 PMC7138021

[130] ElMokh, O., et al. (2022). Gut microbiota severely hampers the efficacy of NAD-lowering therapy in leukemia. *Cell Death & Disease,**13*, Article 320. 35396381 10.1038/s41419-022-04763-3 PMC8993809

[131] Shats, I., et al. (2020). Bacteria boost mammalian host NAD metabolism by engaging the deamidated biosynthesis pathway. *Cell Metabolism,**31*, 564-579.e7. 32130883 10.1016/j.cmet.2020.02.001 PMC7194078

[132] Qiao, K., et al. (2023). Intratumor *Mycoplasma* promotes the initiation and progression of hepatocellular carcinoma. *Cell Reports,**42*, Article 113563. 38088929 10.1016/j.celrep.2023.113563

[133] Kim, J. K., et al. (2023). *Mycoplasma hyorhinis* infection promotes TNF-α signaling and SMAC mimetic-mediated apoptosis in human prostate cancer. *Heliyon,**9*, Article e20655. 37867861 10.1016/j.heliyon.2023.e20655 PMC10585237

[134] Duan, H., et al. (2014). *Mycoplasma hyorhinis* infection promotes NF-κB-dependent migration of gastric cancer cells. *Cancer Research,**74*, 5782–5794. 25136068 10.1158/0008-5472.CAN-14-0650

[135] Basson, C., Serem, J. C., Hlophe, Y. N., & Bipath, P. (2023). The tryptophan–kynurenine pathway in immunomodulation and cancer metastasis. *Cancer Medicine,**12*, 18691–18701. 37644823 10.1002/cam4.6484 PMC10557908

[136] Someya, S., et al. (2021). Tryptophan metabolism regulates proliferative capacity of human pluripotent stem cells. *iScience,**24*, Article 102090. 33615198 10.1016/j.isci.2021.102090 PMC7878994

[137] Qin, R., et al. (2021). Tryptophan potentiates CD8+ T cells against cancer cells by TRIP12 tryptophanylation and surface PD-1 downregulation. *Journal for ImmunoTherapy of Cancer,**9*, Article e002840. 34326168 10.1136/jitc-2021-002840 PMC8323461

[138] Soto-Martin, E. C., et al. (2020). Vitamin biosynthesis by human gut butyrate-producing bacteria and cross-feeding in synthetic microbial communities. *MBio,**11*, e00886-20. 32665271 10.1128/mBio.00886-20 PMC7360928

[139] Ramoneda, J., Jensen, T. B. N., Price, M. N., Casamayor, E. O., & Fierer, N. (2023). Taxonomic and environmental distribution of bacterial amino acid auxotrophies. *Nature Communications,**14*, Article 7608. 37993466 10.1038/s41467-023-43435-4 PMC10665431

[140] Han, K., et al. (2018). Microbiome and butyrate production are altered in the gut of rats fed a glycated fish protein diet. *Journal of Functional Foods,**47*, 423–433.

[141] Van Hul, M., et al. (2020). From correlation to causality: The case of *Subdoligranulum*. *Gut Microbes,**12*, Article 1849998. 33323004 10.1080/19490976.2020.1849998 PMC7744154

[142] Zhou, L., et al. (2018). Faecalibacterium prausnitzii produces butyrate to maintain Th17/Treg balance and to ameliorate colorectal colitis by inhibiting histone deacetylase 1. *Inflammatory Bowel Diseases*, *24*, 1926–1940. 10.1093/ibd/izy182 10.1093/ibd/izy182 29796620

[143] Lin, N.Y.-T., et al. (2025). Microbiota-driven antitumour immunity mediated by dendritic cell migration. *Nature,**644*, 1058–1068. 40659786 10.1038/s41586-025-09249-8 PMC12390848

[144] Kang, X., Lau, H.C.-H., & Yu, J. (2024). Modulating gut microbiome in cancer immunotherapy: Harnessing microbes to enhance treatment efficacy. *Cell Reports Medicine,**5*, Article 101478. 38631285 10.1016/j.xcrm.2024.101478 PMC11031381

[145] Gao, J. et al. (2018). Impact of the gut microbiota on intestinal immunity mediated by tryptophan metabolism. *Frontiers in Cellular and Infection Microbiology*, *8*, Article 13. 10.3389/fcimb.2018.00013 10.3389/fcimb.2018.00013 PMC5808205 29468141

[146] Raudenská, M., et al. (2024). The interplay between microbiome and host factors in pathogenesis and therapy of head and neck cancer. *Biochimica et Biophysica Acta (BBA) - Reviews on Cancer,**1879*, Article 189216. 39542383 10.1016/j.bbcan.2024.189216

[147] Valladares, R., et al. (2013). *Lactobacillus johnsonii* inhibits indoleamine 2,3-dioxygenase and alters tryptophan metabolite levels in BioBreeding rats. *FASEB Journal,**27*, 1711–1720. 23303207 10.1096/fj.12-223339

[148] Fiore, A., et al. (2022). Kynurenine importation by SLC7A11 propagates anti-ferroptotic signaling. *Molecular Cell,**82*, 920-932.e7. 35245456 10.1016/j.molcel.2022.02.007 PMC7617904

[149] Liu, Y., et al. (2018). Tumor-Repopulating Cells Induce PD-1 Expression in CD8+ T Cells by Transferring Kynurenine and AhR Activation. *Cancer Cell,**33*, 480-494.e7. 29533786 10.1016/j.ccell.2018.02.005

[150] Wang, Z., et al. (2025). Kynurenine promotes the immune escape of colorectal cancer cells via NAT10-mediated ac^4^C acetylation of PD-L1. *Clinics (São Paulo, Brazil),**80*, Article 100658. 40245789 10.1016/j.clinsp.2025.100658 PMC12020886

[151] Ostrand-Rosenberg, S., Horn, L. A., & Haile, S. T. (2014). The programmed death-1 immune suppressive pathway: Barrier to anti-tumor immunity. *Journal of Immunology (Baltimore, Md. : 1950),**193*, 3835–3841. 25281753 10.4049/jimmunol.1401572 PMC4185425

[152] Xie, M., et al. (2025). The influence of *Lactobacillus johnsonii* on tumor growth and lymph node metastasis in papillary thyroid carcinoma. *Communications Biology,**8*, Article 419. 40074848 10.1038/s42003-025-07856-9 PMC11903660

[153] Zhou, J., et al. (2024). Tumor-colonized *Streptococcus mutans* metabolically reprograms tumor microenvironment and promotes oral squamous cell carcinoma. *Microbiome,**12*, Article 193. 39369210 10.1186/s40168-024-01907-9 PMC11452938

[154] Sinha, A. K., et al. (2024). Dietary fibre directs microbial tryptophan metabolism via metabolic interactions in the gut microbiota. *Nature Microbiology,**9*, 1964–1978. 38918470 10.1038/s41564-024-01737-3 PMC11306097

[155] Zhou, S., et al. (2025). Indole-3-lactic acid suppresses colorectal cancer via metabolic reprogramming. *Gut Microbes,**17*, 2508949. 40409349 10.1080/19490976.2025.2508949 PMC12118437

[156] Lian, J., et al. (2025). Indole-3-lactic acid inhibits doxorubicin-induced ferroptosis through activating aryl hydrocarbon receptor/Nrf2 signalling pathway. *Journal of Cellular and Molecular Medicine,**29*, Article e70358. 39854052 10.1111/jcmm.70358 PMC11756996

[157] Jia, D., et al. (2024). Microbial metabolite enhances immunotherapy efficacy by modulating T cell stemness in pan-cancer. *Cell,**187*, 1651-1665.e21. 38490195 10.1016/j.cell.2024.02.022

[158] Mu, X., et al. (2025). Decreased gut microbiome-derived indole-3-propionic acid mediates the exacerbation of myocardial ischemia/reperfusion injury following depression via the brain-gut-heart axis. *Redox Biology,**81*, Article 103580. 40058066 10.1016/j.redox.2025.103580 PMC11930714

[159] Han, G., & Lee, D. G. (2022). Indole propionic acid induced Ca2+ -dependent apoptosis in *Candida albicans*. *IUBMB Life,**74*, 235–244. 34779568 10.1002/iub.2579

[160] Wang, D., et al. (2025). Microbiota and cancer: Elucidating the role of *Candida albicans* in cancer progression. *World Journal of Clinical Oncology,**16*, Article 106847. 40585830 10.5306/wjco.v16.i6.106847 PMC12198873

[161] Xu, Y., et al. (2025). Tryptophan metabolic enzyme IL4I1 inhibits ferroptosis by decreasing ubiquitination of Nrf2 via I3P in glioblastoma. *Cell Proliferation,**58*, Article e13816. 40071723 10.1111/cpr.13816 PMC12179557

[162] Jakaria, M., & Cannon, J. R. (2025). The role of hydroxyindoles in protecting neuronal cultures from ferroptosis. *Cell Death Discovery,**11*, Article 329. 40670345 10.1038/s41420-025-02608-4 PMC12267586

[163] Cui, W., et al. (2024). Gut microbial metabolite facilitates colorectal cancer development via ferroptosis inhibition. *Nature Cell Biology,**26*, 124–137. 38168770 10.1038/s41556-023-01314-6

[164] Lee, J., Attila, C., Cirillo, S. L. G., Cirillo, J. D., & Wood, T. K. (2009). Indole and 7-hydroxyindole diminish *Pseudomonas aeruginosa* virulence. *Microbial Biotechnology,**2*, 75–90. 21261883 10.1111/j.1751-7915.2008.00061.x PMC3815423

[165] Li, W., et al. (2025). Gut *Parabacteroides distasonis*-derived indole-3-acetic acid promotes phospholipid remodeling and enhances ferroptosis sensitivity via the AhR-FASN axis in bladder cancer. *Advanced Science,**12*, Article e04688. 40557796 10.1002/advs.202504688 PMC12442663

[166] Pieniazek, A., Bernasinska-Slomczewska, J., & Hikisz, P. (2023). Indoxyl sulfate induces apoptosis in mononuclear blood cells via mitochondrial pathway. *Scientific Reports,**13*, Article 14044. 37640757 10.1038/s41598-023-40824-z PMC10462746

[167] Sun, C.-Y., et al. (2025). Klotho suppresses indoxyl sulfate-mediated apoptosis in human kidney proximal tubular (HK-2) cells through modulating the AKT/Nrf2 mechanism. *ACS Omega,**10*, 24018–24029. 40547630 10.1021/acsomega.4c08038 PMC12177618

[168] Chen, C., et al. (2025). Gut microbiome-derived indole-3-carboxaldehyde regulates stress vulnerability in chronic restraint stress by activating aryl hydrocarbon receptors. *Pharmacological Research,**213*, Article 107654. 39946793 10.1016/j.phrs.2025.107654

[169] Chen, D. Z., Qi, M., Auborn, K. J., & Carter, T. H. (2001). Indole-3-carbinol and diindolylmethane induce apoptosis of human cervical cancer cells and in murine HPV16-transgenic preneoplastic cervical epithelium. *Journal of Nutrition,**131*, 3294–3302. 11739883 10.1093/jn/131.12.3294

[170] Singh, A. A., Jo, S.-H., Kiddane, A. T., Niyonizigiye, I., & Kim, G.-D. (2023). Indole-3-carbinol induces apoptosis in AGS cancer cells via mitochondrial pathway. *Chemical Biology & Drug Design,**101*, 1367–1381. 36798994 10.1111/cbdd.14219

[171] Busbee, P. B., et al. (2020). Indole-3-carbinol prevents colitis and associated microbial dysbiosis in an IL-22–dependent manner. *JCI Insight,**5*, Article e127551. 31941837 10.1172/jci.insight.127551 PMC7030851

[172] Liu, D., et al. (2023). Tryptophan metabolism acts as a new anti-ferroptotic pathway to mediate tumor growth. *Advanced Science,**10*, Article e2204006. 36627132 10.1002/advs.202204006 PMC9951368

[173] Hayashi, T., et al. (2007). 3-Hydroxyanthranilic acid inhibits PDK1 activation and suppresses experimental asthma by inducing T cell apoptosis. *Proceedings of the National Academy of Sciences,**104*, 18619–18624. 10.1073/pnas.0709261104 PMC2141826 18003900

[174] Morita, T., et al. (2001). 3-Hydroxyanthranilic acid, an L-tryptophan metabolite, induces apoptosis in monocyte-derived cells stimulated by interferon-gamma. *Annals of Clinical Biochemistry,**38*, 242–251. 11392499 10.1258/0004563011900461

[175] Jacenik, D., Karagiannidis, I., & Beswick, E. J. (2023). Th2 cells inhibit growth of colon and pancreas cancers by promoting anti-tumorigenic responses from macrophages and eosinophils. *British Journal of Cancer,**128*, 387–397. 36376448 10.1038/s41416-022-02056-2 PMC9902541

[176] Williams, B. B., et al. (2014). Discovery and Characterization of Gut Microbiota Decarboxylases that Can Produce the Neurotransmitter Tryptamine. *Cell Host & Microbe,**16*, 495–503. 25263219 10.1016/j.chom.2014.09.001 PMC4260654

[177] Li, Z., et al. (2022). Dual effect of tryptamine on prostate cancer cell growth regulation: A pilot study. *International Journal of Molecular Sciences,**23*, Article 11087. 36232383 10.3390/ijms231911087 PMC9569450

[178] Dopkins, N., et al. (2021). Tryptamine attenuates experimental multiple sclerosis through activation of aryl hydrocarbon receptor. *Frontiers in Pharmacology*. 10.3389/fphar.2020.619265 33569008 10.3389/fphar.2020.619265 PMC7868334

[179] Herrera, F., et al. (2006). Tryptamine induces cell death with ultrastructural features of autophagy in neurons and glia: Possible relevance for neurodegenerative disorders. *The Anatomical Record. Part A, Discoveries in Molecular, Cellular, and Evolutionary Biology,**288*, 1026–1030. 16892423 10.1002/ar.a.20368

[180] Kim, S., et al. (2024). Oxidative tryptamine dimers from *Corynebacterium durum* directly target survivin to induce AIF-mediated apoptosis in cancer cells. *Biomedicine & Pharmacotherapy,**173*, Article 116335. 38422661 10.1016/j.biopha.2024.116335

[181] Yaku, K., et al. (2012). The enhancement of phase 2 enzyme activities by sodium butyrate in normal intestinal epithelial cells is associated with Nrf2 and p53. *Molecular and Cellular Biochemistry,**370*, 7–14. 22806321 10.1007/s11010-012-1392-x

[182] Donohoe, D. R., et al. (2012). The Warburg effect dictates the mechanism of butyrate-mediated histone acetylation and cell proliferation. *Molecular Cell,**48*, 612–626. 23063526 10.1016/j.molcel.2012.08.033 PMC3513569

[183] Shao, Y., Gao, Z., Marks, P. A., & Jiang, X. (2004). Apoptotic and autophagic cell death induced by histone deacetylase inhibitors. *Proceedings of the National Academy of Sciences,**101*, 18030–18035. 10.1073/pnas.0408345102 PMC539807 15596714

[184] Park, O.-J., et al. (2023). Butyrate potentiates *Enterococcus faecalis* lipoteichoic acid-induced inflammasome activation via histone deacetylase inhibition. *Cell Death Discovery,**9*, Article 107. 36977666 10.1038/s41420-023-01404-2 PMC10050190

[185] Schneewind, O. & Missiakas, D. Lipoteichoic Acid Synthesis and Function in Gram-Positive Bacteria. in *Biogenesis of Fatty Acids, Lipids and Membranes* 1–18 (Springer, Cham, 2017). 10.1007/978-3-319-43676-0_17-2.

[186] Wang, W., et al. (2024). Butyrate and propionate are microbial danger signals that activate the NLRP3 inflammasome in human macrophages upon TLR stimulation. *Cell Reports,**43*, Article 114736. 39277863 10.1016/j.celrep.2024.114736

[187] Chang, C.-C., et al. (2022). Butyrate supplementation regulates expression of chromosome segregation 1‑like protein to reverse the genetic distortion caused by p53 mutations in colorectal cancer. *International Journal of Oncology,**60*, Article 64. 35417036 10.3892/ijo.2022.5354 PMC9084551

[188] Tai, C.-J., Hsu, C.-H., Shen, S.-C., Lee, W.-R., & Jiang, M.-C. (2010). Cellular apoptosis susceptibility (CSE1L/CAS) protein in cancer metastasis and chemotherapeutic drug-induced apoptosis. *Journal of Experimental & Clinical Cancer Research,**29*, Article 110. 20701792 10.1186/1756-9966-29-110 PMC2925819

[189] Alnabulsi, A., et al. (2012). Cellular apoptosis susceptibility (chromosome segregation 1-like, CSE1L) gene is a key regulator of apoptosis, migration and invasion in colorectal cancer. *Journal of Pathology,**228*, 471–481. 22450763 10.1002/path.4031

[190] Wang, Y.-C., et al. (2021). Supplementation of Probiotic Butyricicoccus pullicaecorum Mediates Anticancer Effect on Bladder Urothelial Cells by Regulating Butyrate-Responsive Molecular Signatures. *Diagnostics,**11*, 2270. 34943506 10.3390/diagnostics11122270 PMC8700285

[191] Wang, G., et al. (2023). Butyrate dictates ferroptosis sensitivity through FFAR2-mTOR signaling. *Cell Death & Disease,**14*, 292. 37185889 10.1038/s41419-023-05778-0 PMC10130170

[192] Fu, Q., et al. (2023). Butyrate mitigates metabolic dysfunctions via the ERα-AMPK pathway in muscle in OVX mice with diet-induced obesity. *Cell Communication and Signaling,**21*, 95. 37143096 10.1186/s12964-023-01119-y PMC10158218

[193] He, G., et al. (2014). AMP-activated protein kinase induces p53 by phosphorylating MDMX and inhibiting its activity. *Molecular and Cellular Biology,**34*, 148–157. 24190973 10.1128/MCB.00670-13 PMC3911293

[194] Zhang, T., et al. (2023). Metabolic orchestration of cell death by AMPK-mediated phosphorylation of RIPK1. *Science,**380*, 1372–1380. 37384704 10.1126/science.abn1725 PMC10617018

[195] Hardie, D. G. (2023). Staving off cell death. *Science,**380*, 1322–1323. 37384699 10.1126/science.adi6827

[196] Elangovan, S., et al. (2014). The niacin/butyrate receptor GPR109A suppresses mammary tumorigenesis by inhibiting cell survival. *Cancer Research,**74*, 1166–1178. 24371223 10.1158/0008-5472.CAN-13-1451 PMC3944627

[197] Botta, C., et al. (2022). *Lactiplantibacillus plantarum* inhibits colon cancer cell proliferation as function of its butyrogenic capability. *Biomedicine & Pharmacotherapy,**149*, Article 112755. 35276466 10.1016/j.biopha.2022.112755

[198] Ruemmele, F. M., et al. (1999). Butyrate mediates Caco-2 cell apoptosis via up-regulation of pro-apoptotic BAK and inducing caspase-3 mediated cleavage of poly-(ADP-ribose) polymerase (PARP). *Cell Death and Differentiation,**6*, 729–735. 10467346 10.1038/sj.cdd.4400545

[199] Kim, K., et al. (2019). Propionate of a microbiota metabolite induces cell apoptosis and cell cycle arrest in lung cancer. *Molecular Medicine Reports,**20*, 1569–1574. 31257531 10.3892/mmr.2019.10431 PMC6625448

[200] Liu, X., et al. (2023). Regulation of short-chain fatty acids in the immune system. *Frontiers in Immunology,**14*, 1186892. 37215145 10.3389/fimmu.2023.1186892 PMC10196242

[201] An, J., Kwon, H., & Kim, Y. J. (2023). The Firmicutes/Bacteroidetes ratio as a risk factor of breast cancer. *Journal of Clinical Medicine,**12*, Article 2216. 36983217 10.3390/jcm12062216 PMC10052522

[202] He, T., Cheng, X., & Xing, C. (2021). The gut microbial diversity of colon cancer patients and the clinical significance. *Bioengineered,**12*, 7046–7060. 34551683 10.1080/21655979.2021.1972077 PMC8806656

[203] Schug, Z. T., Voorde, J. V., & Gottlieb, E. (2016). The metabolic fate of acetate in cancer. *Nature Reviews. Cancer,**16*, 708–717. 27562461 10.1038/nrc.2016.87 PMC8992383

[204] Gao, X., et al. (2016). Acetate functions as an epigenetic metabolite to promote lipid synthesis under hypoxia. *Nature Communications,**7*, 11960. 27357947 10.1038/ncomms11960 PMC4931325

[205] Schug, Z. T., et al. (2015). Acetyl-CoA Synthetase 2 Promotes Acetate Utilization and Maintains Cancer Cell Growth under Metabolic Stress. *Cancer Cell,**27*, 57–71. 25584894 10.1016/j.ccell.2014.12.002 PMC4297291

[206] Li, X., et al. (2017). Nucleus-Translocated ACSS2 Promotes Gene Transcription for Lysosomal Biogenesis and Autophagy. *Molecular Cell,**66*, 684-697.e9. 28552616 10.1016/j.molcel.2017.04.026 PMC5521213

[207] Xu, M., et al. (2019). Acetate attenuates inflammasome activation through GPR43-mediated Ca2+-dependent NLRP3 ubiquitination. *Experimental & Molecular Medicine,**51*, 1–13. 10.1038/s12276-019-0276-5 PMC6802670 31337751

[208] Olaniyi, K. S., et al. (2024). Acetate attenuates hypothalamic pyroptosis in experimentally induced polycystic ovarian syndrome. *BMC Research Notes,**17*, Article 260. 39267194 10.1186/s13104-024-06921-6 PMC11395695

[209] Shi, G., et al. (2014). Free fatty acid receptor 2, a candidate target for type 1 diabetes, induces cell apoptosis through ERK signaling. *Journal of Molecular Endocrinology,**53*, 367–380. 25298143 10.1530/JME-14-0065

[210] Mandal, R., et al. (2014). pERK 1/2 inhibit Caspase-8 induced apoptosis in cancer cells by phosphorylating it in a cell cycle specific manner. *Molecular Oncology,**8*, 232–249. 24342355 10.1016/j.molonc.2013.11.003 PMC5528548

[211] Nam, Y. W., et al. (2024). EGFR inhibits TNF-α-mediated pathway by phosphorylating TNFR1 at tyrosine 360 and 401. *Cell Death and Differentiation,**31*, 1318–1332. 38789573 10.1038/s41418-024-01316-3 PMC11445491

[212] Centuori, S. M., et al. (2016). Deoxycholic acid mediates non-canonical EGFR-MAPK activation through the induction of calcium signaling in colon cancer cells. *Biochimica et Biophysica Acta,**1861*, 663–670. 27086143 10.1016/j.bbalip.2016.04.006 PMC4900466

[213] Geismann, C., et al. (2023). NF-κB/RelA controlled A20 limits TRAIL-induced apoptosis in pancreatic cancer. *Cell Death & Disease,**14*, 3. 36596765 10.1038/s41419-022-05535-9 PMC9810737

[214] Chen, S., et al. (2001). WNT-1 signaling inhibits apoptosis by activating β-catenin/T cell factor-mediated transcription. *The Journal of Cell Biology,**152*, 87–96. 11149923 10.1083/jcb.152.1.87 PMC2193656

[215] Chen, J.-Y., et al. (2025). Deoxycholic acid induces reactive oxygen species accumulation and promotes colorectal cancer cell apoptosis through the CaMKII-Ca2+ pathway. *World J Gastrointest Oncol,**17*, Article 107453. 40837770 10.4251/wjgo.v17.i8.107453 PMC12362538

[216] Lanzetti, L. (2024). Oncometabolites at the crossroads of genetic, epigenetic and ecological alterations in cancer. *Cell Death and Differentiation,**31*, 1582–1594. 39438765 10.1038/s41418-024-01402-6 PMC11618380

[217] Humphries, F., et al. (2020). Succination inactivates gasdermin D and blocks pyroptosis. *Science,**369*, 1633–1637. 32820063 10.1126/science.abb9818 PMC8744141

[218] Huang, H., et al. (2025). Succinate accumulation induces pyroptosis and mitochondrial damage via the inhibition of ATP5F1D in HUVECs. *Acta Biochimica et Biophysica Sinica*. 10.3724/abbs.2025116 40817715 10.3724/abbs.2025116 PMC12747930

[219] Cheng, Y., et al. (2024). Downregulation of ATP5F1D inhibits mtROS/NLRP3/caspase-1/GSDMD axis to suppress pyroptosis-mediated malignant progression of endometrial cancer. *International Immunopharmacology,**139*, Article 112808. 39079199 10.1016/j.intimp.2024.112808

[220] Zhao, S., Zhang, Y., Li, C., Zheng, Z., & Ma, M. (2025). Succinate-mediated Ufsp2 transcription promotes high glucose-stimulated pyroptosis in rat retinal Müller cells by activating NLRP3 inflammasome. *Biochemical and Biophysical Research Communications,**783*, Article 152614. 40946554 10.1016/j.bbrc.2025.152614

[221] Zhou, J., et al. (2021). Genomic profiling of the UFMylation family genes identifies UFSP2 as a potential tumour suppressor in colon cancer. *Clinical and Translational Medicine,**11*, Article e642. 34923774 10.1002/ctm2.642 PMC8684770

[222] Lee, S., et al. (2005). Neuronal apoptosis linked to EglN3 prolyl hydroxylase and familial pheochromocytoma genes: Developmental culling and cancer. *Cancer Cell,**8*, 155–167. 16098468 10.1016/j.ccr.2005.06.015

[223] Cai, F., et al. (2024). EGLN3 attenuates gastric cancer cell malignant characteristics by inhibiting JMJD8/NF-κB signalling activation independent of hydroxylase activity. *British Journal of Cancer,**130*, 597–612. 38184692 10.1038/s41416-023-02546-x PMC10876699

[224] Zhao, W., et al. (2024). SLC13A3 is a major effector downstream of activated β-catenin in liver cancer pathogenesis. *Nature Communications,**15*, 7522. 39215042 10.1038/s41467-024-51860-2 PMC11364541

[225] Fernández-Veledo, S., & Vendrell, J. (2019). Gut microbiota-derived succinate: Friend or foe in human metabolic diseases? *Reviews in Endocrine and Metabolic Disorders,**20*, 439–447. 31654259 10.1007/s11154-019-09513-z PMC6938788

[226] Xu, Y. et al. (2020). Function of Akkermansia muciniphila in obesity: Interactions with lipid metabolism, immune response and gut systems. *Frontiers in Microbiology*, *11*, Article 219. 10.3389/fmicb.2020.00219 10.3389/fmicb.2020.00219 PMC7046546 32153527

[227] Chia, L. W., et al. (2018). Deciphering the trophic interaction between Akkermansia muciniphila and the butyrogenic gut commensal Anaerostipes caccae using a metatranscriptomic approach. *Antonie van Leeuwenhoek,**111*, 859–873. 29460206 10.1007/s10482-018-1040-x PMC5945754

[228] Montalban-Arques, A., et al. (2021). Commensal Clostridiales strains mediate effective anti-cancer immune response against solid tumors. *Cell Host & Microbe,**29*, 1573-1588.e7. 34453895 10.1016/j.chom.2021.08.001

[229] Barnett Foster, D., Abul-Milh, M., Huesca, M., & Lingwood, C. A. (2000). Enterohemorrhagic *Escherichia coli* induces apoptosis which augments bacterial binding and phosphatidylethanolamine exposure on the plasma membrane outer leaflet. *Infection and Immunity,**68*, 3108–3115. 10816451 10.1128/iai.68.6.3108-3115.2000 PMC97539

[230] Tang, B., et al. (2015). Shiga toxins induce autophagic cell death in intestinal epithelial cells via the endoplasmic reticulum stress pathway. *Autophagy,**11*, 344–354. 25831014 10.1080/15548627.2015.1023682 PMC4502731

[231] Kuo, C.-J., et al. (2018). A multi-omic analysis reveals the role of fumarate in regulating the virulence of enterohemorrhagic Escherichia coli. *Cell Death & Disease,**9*, 381. 29515100 10.1038/s41419-018-0423-2 PMC5841434

[232] Tauffenberger, A., Fiumelli, H., Almustafa, S., & Magistretti, P. J. (2019). Lactate and pyruvate promote oxidative stress resistance through hormetic ROS signaling. *Cell Death & Disease,**10*, Article 653. 31506428 10.1038/s41419-019-1877-6 PMC6737085

[233] Colbert, L. E., et al. (2023). Tumor-resident Lactobacillus iners confer chemoradiation resistance through lactate-induced metabolic rewiring. *Cancer Cell,**41*, 1945-1962.e11. 37863066 10.1016/j.ccell.2023.09.012 PMC10841640

[234] Yu, H., et al. (2025). Lactate production by tumor-resident *Staphylococcus* promotes metastatic colonization in lung adenocarcinoma. *Cell Host & Microbe,**33*, 1089-1105.e7. 40639336 10.1016/j.chom.2025.06.013

[235] Niu, D., et al. (2025). Lactate prevents glucose deprivation-induced parthanatos in gastric cancer cells through alleviating intracellular reactive oxygen species. *Cell Biology International*. 10.1002/cbin.70064 40745690 10.1002/cbin.70064

[236] Matsuo, T., & Sadzuka, Y. (2018). Extracellular acidification by lactic acid suppresses glucose deprivation-induced cell death and autophagy in B16 melanoma cells. *Biochemical and Biophysical Research Communications,**496*, 1357–1361. 29421654 10.1016/j.bbrc.2018.02.022

[237] Li, C., et al. (2025). Lactylation modification of HIF-1α enhances its stability by blocking VHL recognition. *Cell Communication and Signaling,**23*, Article 364. 40760493 10.1186/s12964-025-02366-x PMC12323271

[238] Chen, Z., Han, F., Du, Y., Shi, H., & Zhou, W. (2023). Hypoxic microenvironment in cancer: Molecular mechanisms and therapeutic interventions. *Signal Transduction and Targeted Therapy,**8*, Article 70. 36797231 10.1038/s41392-023-01332-8 PMC9935926

[239] Yang, Z., et al. (2025). Hypoxia inducible factor-1α drives cancer resistance to cuproptosis. *Cancer Cell,**43*, 937-954.e9. 40054467 10.1016/j.ccell.2025.02.015

[240] Li, H.-S., et al. (2019). HIF-1α protects against oxidative stress by directly targeting mitochondria. *Redox Biology,**25*, Article 101109. 30686776 10.1016/j.redox.2019.101109 PMC6859547

[241] Louis, P., & Flint, H. J. (2017). Formation of propionate and butyrate by the human colonic microbiota. *Environmental Microbiology,**19*, 29–41. 27928878 10.1111/1462-2920.13589

[242] Louis, P., Duncan, S. H., Sheridan, P. O., Walker, A. W., & Flint, H. J. (2022). Microbial lactate utilisation and the stability of the gut microbiome. *Gut Microbiome (Camb),**3*, Article e3. 39295779 10.1017/gmb.2022.3 PMC11406415

[243] Kefayat, A., Bahrami, M., Karami, M., Rostami, S., & Ghahremani, F. (2024). *Veillonella parvula* as an anaerobic lactate-fermenting bacterium for inhibition of tumor growth and metastasis through tumor-specific colonization and decrease of tumor’s lactate level. *Scientific Reports,**14*, Article 21008. 39251652 10.1038/s41598-024-71140-9 PMC11385575

[244] Chang, X., et al. (2023). Propionate-producing Veillonella parvula regulates the malignant properties of tumor cells of OSCC. *Medical Oncology,**40*, 98. 36808012 10.1007/s12032-023-01962-6

[245] Liang, J.-L., et al. (2022). Tailor-made biotuner against colorectal tumor microenvironment to transfer harms into treasures for synergistic oncotherapy. *Nano Today,**47*, Article 101662.

[246] Mardinoglu, A., et al. (2015). The gut microbiota modulates host amino acid and glutathione metabolism in mice. *Molecular Systems Biology,**11*, 834. 26475342 10.15252/msb.20156487 PMC4631205

[247] Dravecka, M., Mikkola, I., Johansen, T., Seternes, O. M., & Mejlvang, J. (2025). Low extracellular pH protects cancer cells from ammonia toxicity. *Cell Death Discovery,**11*, Article 137. 40180899 10.1038/s41420-025-02440-w PMC11968834

[248] Zhang, H., et al. (2024). Ammonia-induced lysosomal and mitochondrial damage causes cell death of effector CD8+ T cells. *Nature Cell Biology,**26*, 1892–1902. 39261719 10.1038/s41556-024-01503-x

[249] Bell, H. N., et al. (2023). Microenvironmental Ammonia Enhances T Cell Exhaustion in Colorectal Cancer. *Cell Metabolism,**35*, 134-149.e6. 36528023 10.1016/j.cmet.2022.11.013 PMC9841369

[250] Jakhar, D., Sarin, S. K., & Kaur, S. (2024). Gut microbiota and dynamics of ammonia metabolism in liver disease. *NPJ Gut and Liver,**1*, Article 11.

[251] Lin, W., et al. (2012). Urease activity represents an alternative pathway for Mycobacterium tuberculosis nitrogen metabolism. *Infection and Immunity,**80*, 2771–2779. 22645285 10.1128/IAI.06195-11 PMC3434571

[252] Nicaise, C., et al. (2008). Control of acute, chronic, and constitutive hyperammonemia by wild-type and genetically engineered Lactobacillus plantarum in rodents. *Hepatology,**48*, 1184–1192. 18697211 10.1002/hep.22445

[253] Nakamura, A., et al. (2021). Symbiotic polyamine metabolism regulates epithelial proliferation and macrophage differentiation in the colon. *Nature Communications,**12*, 2105. 33833232 10.1038/s41467-021-22212-1 PMC8032791

[254] Novita Sari, I., et al*. *(2021). Metabolism and function of polyamines in cancer progression. *Cancer Letters,* *519*, 91–104. 10.1016/j.canlet.2021.06.020 34186159

[255] Chen, H., et al. (2023). Urea cycle activation triggered by host-microbiota maladaptation driving colorectal tumorigenesis. *Cell Metabolism,**35*, 651-666.e7. 36963394 10.1016/j.cmet.2023.03.003

[256] Xu, S. et al. (2025). Intestinal microbiota affects the progression of colorectal cancer by participating in the host intestinal arginine catabolism. *Cell Reports*, *44*, Article 115370. 10.1016/j.celrep.2025.115370 10.1016/j.celrep.2025.115370 40022728

[257] Fan, L., et al. (2024). Intestinal *Lactobacillus murinus*-derived small RNAs target porcine polyamine metabolism. *Proceedings of the National Academy of Sciences of the United States of America,**121*, Article e2413241121. 39361652 10.1073/pnas.2413241121 PMC11474053

[258] Murray Stewart, T., Dunston, T. T., Woster, P. M., & Casero, R. A. (2018). Polyamine catabolism and oxidative damage. *Journal of Biological Chemistry,**293*, 18736–18745. 30333229 10.1074/jbc.TM118.003337 PMC6290137

[259] Affronti, H. C., et al. (2020). Pharmacological polyamine catabolism upregulation with methionine salvage pathway inhibition as an effective prostate cancer therapy. *Nature Communications,**11*, 52. 31911608 10.1038/s41467-019-13950-4 PMC6946658

[260] Bi, G., et al. (2024). Polyamine-mediated ferroptosis amplification acts as a targetable vulnerability in cancer. *Nature Communications,**15*, 2461. 38504107 10.1038/s41467-024-46776-w PMC10951362

[261] Pietzke, M., Meiser, J., & Vazquez, A. (2019). Formate metabolism in health and disease. *Molecular Metabolism,**33*, 23–37. 31402327 10.1016/j.molmet.2019.05.012 PMC7056922

[262] Yu, S. Y., et al. (2019). Bacteroides faecalis sp. nov., isolated from human faeces. *International Journal of Systematic and Evolutionary Microbiology*, *69*, 3824–3829. 10.1099/ijsem.0.003690 31511127

[263] Wang, M., Wang, Z., Lessing, D. J., Guo, M., & Chu, W. (2023). *Fusobacterium nucleatum* and its metabolite hydrogen sulfide alter gut microbiota composition and autophagy process and promote colorectal cancer progression. *Microbiology Spectrum*. 10.1128/spectrum.02292-23 37889013 10.1128/spectrum.02292-23 PMC10714730

[264] Lin, H., et al. (2023). Implications of hydrogen sulfide in colorectal cancer: Mechanistic insights and diagnostic and therapeutic strategies. *Redox Biology,**59*, Article 102601. 36630819 10.1016/j.redox.2023.102601 PMC9841368

[265] Pidgeon, R., et al. (2025). Diet-derived urolithin A is produced by a dehydroxylase encoded by human gut Enterocloster species. *Nature Communications,**16*, 999. 39856097 10.1038/s41467-025-56266-2 PMC11760930

[266] El-Wetidy, M. S., et al. (2021). Urolithin A induces cell cycle arrest and apoptosis by inhibiting Bcl-2, increasing p53–p21 proteins and reactive oxygen species production in colorectal cancer cells. *Cell Stress and Chaperones,**26*, 473–493. 33666815 10.1007/s12192-020-01189-8 PMC8065090

[267] Mohammed Saleem, Y. I., Albassam, H., & Selim, M. (2020). Urolithin A induces prostate cancer cell death in p53-dependent and in p53-independent manner. *European Journal of Nutrition,**59*, 1607–1618. 31177307 10.1007/s00394-019-02016-2

[268] Zheng, B., et al. (2024). Urolithin A inhibits breast cancer progression via activating TFEB-mediated mitophagy in tumor macrophages. *Journal of Advanced Research,**69*, 125–138. 38615740 10.1016/j.jare.2024.04.010 PMC11954813

[269] Yu, T., et al. (2017). *Fusobacterium nucleatum* Promotes Chemoresistance to Colorectal Cancer by Modulating Autophagy. *Cell,**170*, 548-563.e16. 28753429 10.1016/j.cell.2017.07.008 PMC5767127

[270] Huang, P., et al. (2024). Peptostreptococcus stomatis promotes colonic tumorigenesis and receptor tyrosine kinase inhibitor resistance by activating ERBB2-MAPK. *Cell Host & Microbe,**32*, 1365-1379.e10. 39059397 10.1016/j.chom.2024.07.001

[271] Geller, L. T., et al. (2017). Potential role of intratumor bacteria in mediating tumor resistance to the chemotherapeutic drug gemcitabine. *Science,**357*, 1156–1160. 28912244 10.1126/science.aah5043 PMC5727343

[272] Colbert, L. E., et al. (2023). Tumor-resident *Lactobacillus iners* confer chemoradiation resistance through lactate-induced metabolic rewiring. *Cancer Cell,**41*, 1945-1962.e11. 37863066 10.1016/j.ccell.2023.09.012 PMC10841640

[273] Battaglia, T. W., et al. (2024). A pan-cancer analysis of the microbiome in metastatic cancer. *Cell,**187*, 2324-2335.e19. 38599211 10.1016/j.cell.2024.03.021

[274] Li, J., Qin, Y., Zhao, C., Zhang, Z., & Zhou, Z. (2023). Tetracycline antibiotics: Potential anticancer drugs. *European Journal of Pharmacology,**956*, Article 175949. 37541377 10.1016/j.ejphar.2023.175949

[275] Bullman, S., et al. (2017). Analysis of Fusobacterium persistence and antibiotic response in colorectal cancer. *Science,**358*, 1443–1448. 29170280 10.1126/science.aal5240 PMC5823247

[276] See, W. A., et al. (2009). Bacille-Calmette Guèrin induces caspase-independent cell death in urothelial carcinoma cells together with release of the necrosis-associated chemokine high molecular group box protein 1. *BJU International,**103*, 1714–1720. 19154459 10.1111/j.1464-410X.2008.08274.x

[277] Tadie, J.-M., et al. (2013). HMGB1 promotes neutrophil extracellular trap formation through interactions with Toll-like receptor 4. *American Journal of Physiology-Lung Cellular and Molecular Physiology,**304*, L342–L349. 23316068 10.1152/ajplung.00151.2012 PMC3602738

[278] Theys, J., Patterson, A. V., & Mowday, A. M. (2024). *Clostridium* bacteria: Harnessing tumour necrosis for targeted gene delivery. *Molecular Diagnosis & Therapy,**28*, 141–151. 38302842 10.1007/s40291-024-00695-0 PMC10925577

[279] Meng, Y., et al. (2024). Pyroptosis regulation by Salmonella effectors. *Frontiers in Immunology*, *15*, Article 1464858. 10.3389/fimmu.2024.1464858 10.3389/fimmu.2024.1464858 PMC11538000 39507539

[280] Pérez Jorge, G., Gontijo, M. T. P., & Brocchi, M. (2023). *Salmonella enterica* and outer membrane vesicles are current and future options for cancer treatment. *Frontiers in Cellular and Infection Microbiology,**13*, Article 1293351. 38116133 10.3389/fcimb.2023.1293351 PMC10728604

[281] Mercado-Lubo, R., et al. (2016). A *Salmonella* nanoparticle mimic overcomes multidrug resistance in tumours. *Nature Communications,**7*, Article 12225. 27452236 10.1038/ncomms12225 PMC5512628

[282] Nemunaitis, J., et al*.* (2003). Pilot trial of genetically modified, attenuated Salmonella expressing the E. coli cytosine deaminase gene in refractory cancer patients. *Cancer Gene Ther*, *10*, 737–744. 10.1038/sj.cgt.7700634 14502226

[283] Yoon, W., Yoo, Y., Chae, Y. S., Kee, S.-H., & Kim, B. M. (2018). Therapeutic advantage of genetically engineered *Salmonella typhimurium* carrying short hairpin RNA against inhibin alpha subunit in cancer treatment. *Annals of Oncology,**29*, 2010–2017. 30016386 10.1093/annonc/mdy240

[284] Copland, A., et al. (2024). *Salmonella* cancer therapy metabolically disrupts tumours at the collateral cost of T cell immunity. *EMBO Molecular Medicine,**16*, 3057–3088. 39558103 10.1038/s44321-024-00159-2 PMC11628626

[285] Cooper, R. M., et al. (2023). Engineered bacteria detect tumor DNA. *Science,**381*, 682–686. 37561843 10.1126/science.adf3974 PMC10852993

[286] Sheng, D., et al. (2024). Pan-cancer atlas of tumor-resident microbiome, immunity and prognosis. *Cancer Letters,**598*, Article 217077. 38908541 10.1016/j.canlet.2024.217077

[287] Blount, Z. D. (2015). The unexhausted potential of E. coli. *eLife*, *4*, e05826. 10.7554/eLife.05826 PMC4373459 25807083

[288] Sewell, A. K., et al. (2025). Enterobactin carries iron into Caenorhabditis elegans and mammalian intestinal cells by a mechanism independent of divalent metal transporter DMT1. *Journal of Biological Chemistry*, *301*, Article 108158. 10.1016/j.jbc.2025.108158 10.1016/j.jbc.2025.108158 PMC11815940 39761858

[289] Chi, Y., et al. (2020). Cancer cells deploy lipocalin-2 to collect limiting iron in leptomeningeal metastasis. *Science,**369*, 276–282. 32675368 10.1126/science.aaz2193 PMC7816199

[290] Qi, B., & Han, M. (2018). Microbial siderophore enterobactin promotes mitochondrial iron uptake and development of the host via interaction with ATP synthase. *Cell,**175*, 571-582.e11. 30146159 10.1016/j.cell.2018.07.032

[291] Xiong, L., et al. (2020). Ahr-Foxp3-RORγt axis controls gut homing of CD4+ T cells by regulating GPR15. *Science Immunology*, *5*, eaaz7277. 10.1126/sciimmunol.aaz7277 PMC7371246 32532834

[292] Pollet, M., et al. (2018). The AHR represses nucleotide excision repair and apoptosis and contributes to UV-induced skin carcinogenesis. *Cell Death and Differentiation,**25*, 1823–1836. 30013037 10.1038/s41418-018-0160-1 PMC6180092

[293] Kou, Z., et al. (2024). AhR signaling modulates ferroptosis by regulating SLC7A11 expression. *Toxicology and Applied Pharmacology,**486*, Article 116936. 38641223 10.1016/j.taap.2024.116936

[294] Foley, S. E., et al. (2021). Gut microbiota regulation of P-glycoprotein in the intestinal epithelium in maintenance of homeostasis. *Microbiome,**9*, Article 183. 34493329 10.1186/s40168-021-01137-3 PMC8425172

[295] Xiao, H., et al. (2020). Gut microbiota-derived indole 3-propionic acid protects against radiation toxicity via retaining acyl-CoA-binding protein. *Microbiome,**8*, 69. 32434586 10.1186/s40168-020-00845-6 PMC7241002

[296] Wang, M., et al. (2024). Killing tumor-associated bacteria with a liposomal antibiotic generates neoantigens that induce anti-tumor immune responses. *Nature Biotechnology,**42*, 1263–1274. 37749267 10.1038/s41587-023-01957-8 PMC12892252

[297] Ahmad, N., et al. (2024). Nanoparticles incorporated hydrogels for delivery of antimicrobial agents: Developments and trends. *RSC Advances,**14*, 13535–13564. 38665493 10.1039/d4ra00631c PMC11043667

[298] Zhang, X., et al. (2021). Dual gate-controlled therapeutics for overcoming bacterium-induced drug resistance and potentiating cancer immunotherapy. *Angewandte Chemie (International ed. in English),**60*, 14013–14021. 33768682 10.1002/anie.202102059

[299] Liping, Z., et al. (2024). Comprehensive retrospect and future perspective on bacteriophage and cancer. *Virology Journal,**21*, 278. 39501333 10.1186/s12985-024-02553-1 PMC11539450

[300] Vincent, R. L., et al. (2023). Probiotic-guided CAR-T cells for solid tumor targeting. *Science,**382*, 211–218. 37824640 10.1126/science.add7034 PMC10915968

[301] Chiang, C.-J., & Hong, Y.-H. (2021). In situ delivery of biobutyrate by probiotic *Escherichia coli* for cancer therapy. *Scientific Reports,**11*, Article 18172. 34518590 10.1038/s41598-021-97457-3 PMC8438071

[302] Li, Y., et al. (2024). Microbial metabolite sodium butyrate enhances the anti-tumor efficacy of 5-fluorouracil against colorectal cancer by modulating PINK1/Parkin signaling and intestinal flora. *Scientific Reports,**14*, Article 13063. 38844824 10.1038/s41598-024-63993-x PMC11156851

[303] Tintelnot, J., et al. (2023). Microbiota-derived 3-IAA influences chemotherapy efficacy in pancreatic cancer. *Nature,**615*, 168–174. 36813961 10.1038/s41586-023-05728-y PMC9977685

[304] Dong, J., et al. (2024). Roseburia intestinalis sensitizes colorectal cancer to radiotherapy through the butyrate/OR51E1/RALB axis. *Cell Reports,**43*, Article 113846. 38412097 10.1016/j.celrep.2024.113846

[305] Wu, N., Wei, X., Yu, S., Yang, L., & Zhang, X. (2025). Lactate in ferroptosis regulation: A new perspective on tumor progression and therapy. *Pharmacological Research,**218*, Article 107841. 40582630 10.1016/j.phrs.2025.107841

